# Split-Dose Cisplatin Use, Eligibility Criteria, and Drivers for Treatment Choice in Patients with Locally Advanced or Metastatic Urothelial Carcinoma: Results of a Large International Physician Survey

**DOI:** 10.3390/cancers17030509

**Published:** 2025-02-03

**Authors:** Richard O’Dwyer, Sophia Junker, Robert Szulkin, Scarlette Kienzle, Mairead Kearney, Srikala S. Sridhar

**Affiliations:** 1 Division of Medical Oncology, Princess Margaret Cancer Centre, University of Toronto, 610 University Avenue, Toronto, ON M5G 2M9, Canada; richardtodwyer@gmail.com; 2 Cytel Inc., Potsdamer Strasse 58, 7th Floor, 10785 Berlin, Germany; sophia.junker@outlook.com (S.J.); scarlette.kienzle@cytel.com (S.K.); 3 Cytel Inc., Sankt Eriksgatan 113, 3rd Floor, 113 43 Stockholm, Sweden; robert.szulkin@cytel.com; 4 Merck Healthcare KGaA, Frankfurter Strasse 250, 64293 Darmstadt, Germany; mairead.kearney@merckgroup.com

**Keywords:** split-dose cisplatin, advanced urothelial cancer, physician survey, international, real-world evidence

## Abstract

Background: For many decades, gemcitabine + cisplatin has been a preferred and accepted treatment option for patients with urothelial cancer (UC). In patients ineligible for standard-dose cisplatin, split-dose cisplatin is a promising alternative. This study aimed to provide insights into the use of split-dose cisplatin and factors influencing treatment choice. Methods: Between January and March 2024, an international cross-sectional survey was carried out, which involved oncologists and urologists treating patients with locally advanced/metastatic UC (la/mUC) in Australia, Brazil, Canada, France, Germany, India, Italy, Spain, the UK, and the USA. Demographics, practice patterns, and clinical parameters influencing treatment choice were collected. Results: Of the 791 respondents, most were male (73%), the mean age was 43 years, and the mean time spent in clinical practice was 13 years. In total, 85% reported using split-dose cisplatin in UC, ranging from 97% in Canada to 67% in Brazil. The preferred schedule in la/mUC was gemcitabine + cisplatin 35 mg/m^2^ on days 1 and 8 of 21-day cycles (57%). Most respondents (64%) were comfortable prescribing split-dose cisplatin to otherwise fit patients with a creatinine clearance ≥40 mL/min. Standard- and split-dose cisplatin were preferred regimens for otherwise fit patients with creatinine clearance of 45–60 mL/min. Conclusions: This large international survey demonstrates the extensive use of split-dose cisplatin in patients with la/mUC. Responses indicate that split-dose cisplatin is administered to patients in clinical practice with a wider range of creatinine clearance, performance status, and comorbidities than suggested for standard-dose cisplatin. Results highlight the need to evaluate split-dose cisplatin prospectively and establish consensus guidelines for its use, especially in patients unfit for standard-dose cisplatin.

## Simple Summary

In total, 791 oncologists/urologists in 10 countries were surveyed about their use of split-dose cisplatin treatment in patients with urothelial carcinoma (UC) in routine clinical practice. Overall, 85% of physicians reported using split-dose cisplatin for the treatment of UC. In all countries, respondents reported using split-dose cisplatin in patients with locally advanced or metastatic UC across a range of creatinine clearance levels and comorbidity grades. To our knowledge, this is the first international survey on the use of split-dose cisplatin in UC. These results highlight a need to establish consensus guidelines for optimal use of split-dose cisplatin in clinical practice.

## 1. Introduction

One of the most important regimens in managing urothelial cancer (UC) consists of a combination of gemcitabine and cisplatin (GC) [1]. In muscle-invasive bladder cancer, GC is the standard of care in the neoadjuvant setting, and until recently, this regimen was the preferred first-line (1L) treatment option in cisplatin-eligible patients with locally advanced or metastatic UC (la/mUC). Patients without disease progression on 1L GC are recommended to receive maintenance avelumab [2,3,4,5,6,7]. GC has also been shown to combine favorably with an immune checkpoint inhibitor (ICI) in a recent phase 3 1L study, suggesting that GC may also have important immunomodulatory effects [8]. In the standard GC regimen, cisplatin is administered on day 1 of a 3-week cycle at a dose of 70 mg/m^2^, and gemcitabine is administered on days 1 and 8 at a dose of 1250 mg/m^2^.

Unfortunately, many patients with la/mUC are not candidates for standard cisplatin-based treatment due to comorbidities [9,10]. In 2011, Galsky et al. published criteria for cisplatin ineligibility based on a survey of 120 international genitourinary medical oncologists [10]. The classification includes Eastern Cooperative Oncology Group performance status (ECOG PS) ≥2, creatinine clearance (CrCl) ≤60 mL/min, audiometric hearing loss grade ≥2, peripheral neuropathy (PN) grade ≥2, and/or heart failure based on New York Heart Association (NYHA) class ≥III. These thresholds were endorsed by guidelines from the European Society for Medical Oncology (ESMO) and the European Association of Urology (EAU) [2,3,4].

In patients deemed cisplatin ineligible, carboplatin can be offered but may result in inferior outcomes [11]. There is therefore significant interest in exploring strategies that could allow more patients to receive cisplatin while minimizing toxicity. One frequently used approach is a split-dose cisplatin regimen, in which the cisplatin dose is divided over 2 days, with the aim of maintaining overall dose intensity but minimizing toxicity [12,13]. Several studies have suggested that split-dose cisplatin regimens may be an effective alternative to standard-dose cisplatin both in the neoadjuvant and in the la/mUC settings; data have shown a higher objective response rate with split-dose cisplatin regimens compared with gemcitabine + carboplatin (GCa), and similar overall survival to both GC and GCa [13,14,15,16]. EAU, ESMO, and the National Comprehensive Cancer Network (NCCN) guidelines for UC support the use of split-dose cisplatin regimens in specific situations [2,3,4,6].

Despite encouraging results with split-dose cisplatin, clear and uniform guidance on its use, in addition to real-world data from different healthcare settings, is lacking. Currently, patients deemed cisplatin ineligible by Galsky criteria may not be considered for any cisplatin-based therapy [17]. Classifying patients as cisplatin ineligible when a split-dose regimen is feasible could result in some patients being undertreated.

The aim of this study was to survey a representative sample of physicians across 10 countries to describe the frequency of split-dose cisplatin use in the treatment of UC, focusing specifically on la/mUC. We also sought to better understand patient and clinical characteristics that physicians consider when making decisions to prescribe split-dose cisplatin for patients with la/mUC in routine clinical practice, and to identify factors that influence the choice of a split-dose cisplatin-based approach.

## 2. Methods

### 2.1. Study Design

This was an international, cross-sectional, anonymous online survey of physicians, designed in collaboration with expert clinicians to collect information on the current use and perceptions of split-dose cisplatin for the treatment of UC. The English-language survey draft was translated into local languages by medical linguists in a two-step process. To validate the questionnaire, qualitative pilot interviews lasting 60 min were conducted with two physicians per country between 27 September and 3 November 2023. These interviews focused on evaluating the relevance, completeness, clarity, and applicability of the survey questions to each country, and the adequacy of the planned quotas for the respective countries. The questionnaire was edited based on the pilot feedback, and the final questionnaire can be found in Supplemental Table S1.

After the pilot interviews and finalization of the survey, data were collected anonymously via a secure online data collection platform. Several measures were taken to ensure data quality, including the use of predefined answer options wherever applicable; automatic answer verification via plausibility checks; quality review of data exports at 10%, 50%, and 100% of data collection; and exclusion of respondents with conspicuous answers (e.g., selecting the same answer number for multiple items in a row, completing the questionnaire irregularly quickly). In both phases of data collection, respondents were masked to the study sponsor, and the study sponsor was masked to the respondents.

### 2.2. Study Participants

Physicians were recruited between January and March 2024 in 10 countries of interest (EU5 [France, Germany, Italy, Spain, and the UK], USA, India, Brazil, Canada, and Australia) via a proprietary provider panel (M3 Difference Panel by M3 Global Research). Physicians underwent three-factor verification of their identities, qualifications, and areas of specialty upon panel admittance. Requirements for study eligibility included specialization as a medical oncologist or urologist in the respective country (urologists were eligible to participate in India, Germany, and Spain only because they are common prescribers of systemic anticancer treatment for UC in these countries), ≥2 years of clinical experience after the end of their medical specialist training, treatment of ≥10 patients with UC during the past year, treatment of ≥5 patients with la/mUC during the past year, and large or full responsibility for prescribing and overseeing systemic anticancer therapy.

The study protocol of this research was reviewed by the Salus Institutional Review Board and received exemption status from the need for institutional review board review on 8 September 2023. All participants agreed to the M3 Global Research terms of use and privacy policy upon entering the panel and acknowledged receipt of an information statement at the start of the survey.

### 2.3. Data Collection

A study invitation was sent to verified medical oncologists and urologists who previously indicated that they treat UC, which included general information about the study (length of engagement and amount of honorarium). To minimize response bias, inclusion criteria and information about the questionnaire’s purpose were not included in the initial reach-out. Only respondents who met all criteria were directed to the full questionnaire and received monetary compensation. The information statement at the start of the survey specifically encouraged physicians to continue the survey irrespective of whether they used split-dose cisplatin in clinical practice.

Quotas were applied with the aim of recruiting a cohort of respondents that would be representative of the physician landscape treating la/mUC with systemic anticancer therapy within each country in terms of primary practice location (academic or teaching hospital/specialized cancer center, community/nonteaching hospital, office-based practice) and medical specialty (medical oncologist, urologist).

### 2.4. Statistical Analysis

Responses to survey items were descriptively analyzed overall and individually by country. Categorical variables were described by the total respondent number and percentages. Continuous data were summarized using mean, standard deviation, and/or median and interquartile range. Responses were additionally analyzed by geographic region (Europe [EU5], North America [USA, Canada], Brazil, India, and Australia), with statistical comparisons conducted using the Kruskal-Wallis test for continuous variables and the Chi-square test for categorical variables. A multivariable logistic regression model was used to assess the factors exerting an influence on prescribing behavior for split-dose cisplatin across countries. Variables for the final model were selected using the backward elimination technique; initial variables included specialty, number of practice years, level of responsibility in prescribing systemic anticancer therapy, practice setting, practice location, gender, enrollment of patients with UC in clinical trials, and total number of patients with UC treated per month. Univariate analyses were performed for these initial variables. Analyses were conducted using Excel (Microsoft; Redmond, WA, USA) and RStudio version 4.3.3 (Posit; Boston, MA, USA) [18].

## 3. Results

### 3.1. Physician Demographics and Characteristics

Of 1254 physicians who entered the screening process, 791 met all eligibility criteria and completed the survey (USA, 151; India, 107; Brazil, 100; EU5 countries, 75 each; Canada, 33; and Australia, 25; see Supplemental Table S2 for selection criteria and reasons for exclusion). Physician demographics and characteristics varied significantly across geographic regions for most attributes (Table 1). Participant demographics and characteristics by individual country are provided in Supplemental Table S3. Participants comprised medical oncologists (92%) and urologists (8%), 73% were male, the mean age was 43 years, and the mean length of clinical practice experience was 13 years. Most respondents were based in an academic or teaching hospital or a specialized care center (59%), followed by a community or nonteaching hospital (27%) and an office-based practice (14%). Participants were mainly recruited from large hospitals (51% of all respondents) and were based in the public setting (55%). Respondents treated a mean of 42 patients with UC per month (including first-time and follow-up patients; median, 25). The average monthly la/mUC patient load was 28 (median, 15). Approximately half of the respondents enrolled patients with UC in clinical trials.

Participants considered a variety of guidelines for the treatment of UC. The guideline published by the NCCN was the most popular (62%), followed by ESMO (60%) and the American Society of Clinical Oncology guidelines (48%). While the ESMO guideline was often referred to in France, the UK, Italy, and Spain (80–91%), the NCCN guideline was more often referred to in non-European countries (67–87%). Respondents used a variety of methods to assess kidney function; calculated CrCl was the most common method across countries (86%), most often computed using the Cockcroft-Gault equation (68%).

### 3.2. Use of Split-Dose Cisplatin in UC and la/mUC

Of 791 respondents, 670 (85%) reported using split-dose cisplatin regimens in patients with UC (Table 1). Physicians in Canada had the highest rate of prescribing split-dose cisplatin (97%), while Brazil (67%) and India (77%) reported the lowest rates (Supplemental Table S3). Respondents who indicated using split-dose cisplatin prescribed it to a similar proportion of patients in each queried setting (neoadjuvant: mean, 41% [median, 34]; adjuvant: mean, 39% [median, 35]; advanced: mean, 43% [median, 40]).

Physicians who indicated prescribing split-dose cisplatin to patients with la/mUC (n = 660) were asked for further details on its use in la/mUC (Table 2). The majority considered split-dose cisplatin for 1L treatment (83%) and approximately half considered it for the second line. The most frequently used split-dose cisplatin regimen was cisplatin 35 mg/m^2^ in combination with gemcitabine given on days 1 and 8 of a 21-day cycle (57%). More than 80% of physicians reported routinely using avelumab as 1L maintenance in la/mUC if no disease progression occurred during or after split-dose cisplatin-based chemotherapy. The use of avelumab as a 1L maintenance treatment in la/mUC was reported by >75% of physicians in all countries except India (28%). The use of split-dose cisplatin across treatment lines (1L, 2L, 3L) and the use of 1L avelumab maintenance treatment in patients with la/mUC showed significant variation by geographic region, whereas no significant differences in specific split-dose cisplatin regimens were observed (Supplemental Table S4).

Overall, 121 respondents (15%) indicated that they never used split-dose cisplatin regimens. The most common reasons cited were: the regimen not being part of institutional treatment protocols or guidelines (45%), lack of evidence for efficacy (31%), and concerns regarding toxicity in patients with poor renal function (28%) (Supplemental Table S5).

### 3.3. Physician Characteristics Influencing Prescribing Behavior for Split-Dose Cisplatin

In the multivariable analysis conducted across all countries, excluding geographic region as a covariate, several physician characteristics were associated with a greater likelihood of prescribing split-dose cisplatin to patients with UC: longer time in practice (per decade: odds ratio [OR], 1.58; 95% CI, 1.18–2.16); higher patient volume (per 10 patients: OR, 1.06; 95% CI, 1.01–1.14); public vs. private practice setting (OR, 1.94; 95% CI, 1.27–2.97); spending equal time in the public and private settings vs. the private setting alone (OR, 2.58; 95% CI, 1.21–6.17); and oncologist vs. urologist specialty (OR, 0.45; 95% CI, 0.24–0.88) (Table 3). When the geographic region was included as a covariate, physicians were significantly less likely to prescribe split-dose cisplatin in Brazil (OR, 0.30; 95% CI, 0.14–0.64) compared with physicians in Europe, and practice setting and higher patient volume were no longer significant factors. In univariate analyses, a higher likelihood of prescribing split-dose cisplatin was associated with longer time in practice, working in a public setting or spending equal time in public and private settings, and higher patient volume, whereas physicians from Brazil and India were less likely to prescribe split-dose cisplatin compared with respondents from Europe (Supplementary Table S6). Stratifying the multivariate analyses by geographical region, with region excluded as a covariate, revealed that prescribing split-dose cisplatin was associated with longer time in practice in Europe (OR 1.78; 95% CI, 1.06–3.10) and oncologist vs. urologist specialty in India (OR 0.09; 95% CI, 0.02–0.34), with no other covariates reaching significance (Supplementary Table S7).

### 3.4. Eligibility Criteria and Thresholds for the Use of Split-Dose Cisplatin in la/mUC

**Figure 1:**
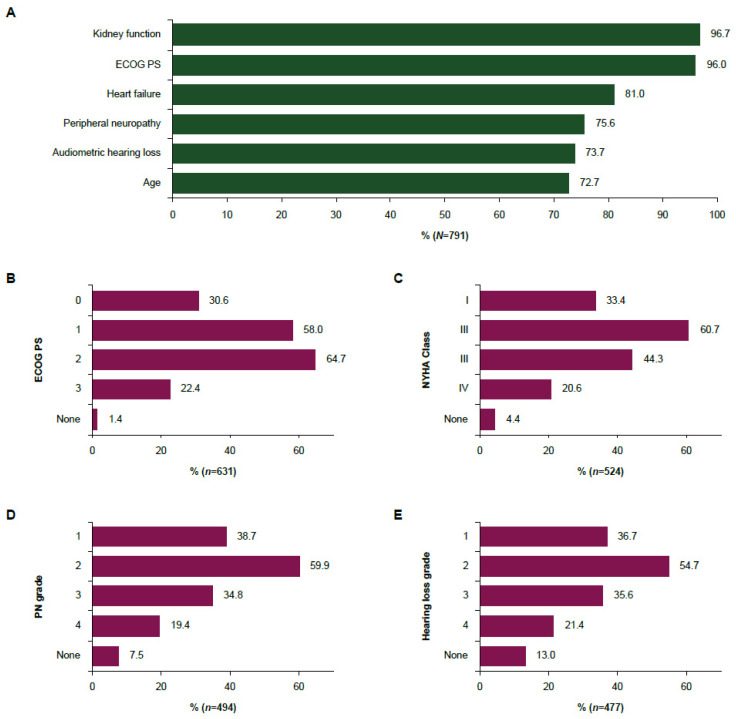
Consideration of characteristics in physician’s decision to prescribe (**A**) platinum-based chemotherapy and (**B**–**E**) split-dose cisplatin. (**A**) Percentage of physicians who consider a characteristic relevant when deciding on the type of platinum-based chemotherapy in an otherwise fit patient with la/mUC. (**B**) ECOG PS scores, (**C**) heart failure grades, (**D**) PN grades, and (**E**) audiometric hearing loss grades chosen for treatment with split-dose cisplatin rather than standard-dose cisplatin in an otherwise fit patient with la/mUC (multiple answers possible). For (**B**–**E**), n was based on respondents who indicated finding the respective characteristic relevant as shown in (**A**). None indicates “I would not prescribe split-dose cisplatin in any of these scenarios”. ECOG PS, Eastern Cooperative Oncology Group performance status; la/mUC, locally advanced or metastatic urothelial cancer; NYHA, New York Heart Association; PN, peripheral neuropathy.

All participants were asked whether they considered any of six characteristics when deciding on the type of platinum-based chemotherapy in an otherwise fit patient with la/mUC (defined as ECOG PS 0–1 without other comorbidities affecting treatment decisions; questions considering ECOG PS did not contain any specifications regarding ECOG PS or comorbidity status of the patient.). Almost all respondents considered ECOG PS (97%) and kidney function (96%) in their decision (Figure 1A). Heart failure was considered by 81% of respondents, and PN, audiometric hearing loss, and age were each considered by approximately three-quarters of respondents.

Respondents who indicated prescribing split-dose cisplatin in la/mUC and reported considering each characteristic were asked to provide further details on the scores, grades, and thresholds they would choose for prescribing split-dose cisplatin instead of standard-dose cisplatin to an otherwise fit patient with la/mUC (Figure 1B–E). The most common choices were ECOG PS 1 (59%) and 2 (65%), NYHA grade II (61%) and III (44%), PN grade 1 (39%) and 2 (60%), and audiometric hearing loss grade 1 (37%) and 2 (55%).

**Figure 2:**
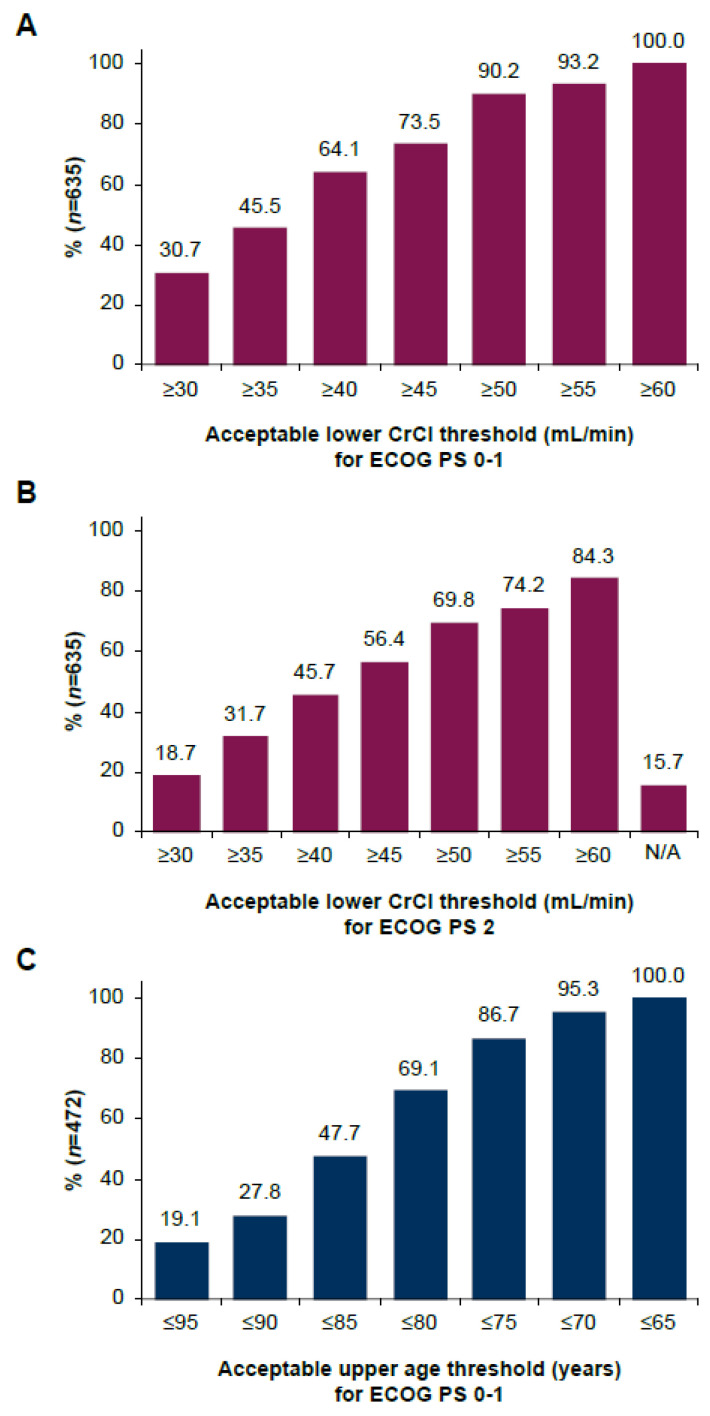
Consideration of characteristics in physician’s decision to prescribe split-dose cisplatin. (**A**) Acceptable lower creatinine clearance thresholds (mL/min) for an otherwise fit patient with la/mUC and ECOG PS 0–1 (percentage of physicians who selected the respective threshold or lower). (**B**) Acceptable lower CrCl thresholds (mL/min) for a patient with la/mUC and ECOG PS 2 (percentage of physicians who selected the respective threshold or lower). (**C**) Acceptable upper age thresholds in years for an otherwise fit patient with la/mUC and ECOG PS 0–1 (percentage of physicians who selected the respective threshold or higher). CrCl, creatinine clearance; ECOG PS, Eastern Cooperative Oncology Group performance status; la/mUC, locally advanced or metastatic urothelial cancer. N was based on respondents who indicated finding the respective characteristic relevant as shown in Figure 1A. N/A indicates “I would not prescribe any cisplatin regimen in a patient with ECOG PS 2”.

A majority of respondents were comfortable using split-dose cisplatin in an otherwise fit patient with la/mUC with a CrCl of ≥40 mL/min (64%), whereas 90% were comfortable with a threshold of ≥50 mL/min (Figure 2A). When respondents were asked about use in a patient with ECOG PS 2, percentages dropped to 46% for CrCl ≥40 mL/min and 70% for CrCl ≥50 mL/min. Overall, 16% of respondents indicated that they would not prescribe any cisplatin regimen to a patient with ECOG PS 2 (Figure 2B). Almost 70% were comfortable prescribing split-dose cisplatin to otherwise fit patients with la/mUC who were aged ≥80 years (Figure 2C). Consideration of these characteristics in the decision to prescribe split-dose cisplatin differed significantly by geographical region (Supplementary Table S8).

### 3.5. Treatment Preferences for la/mUC with Borderline Kidney Function

**Figure 3:**
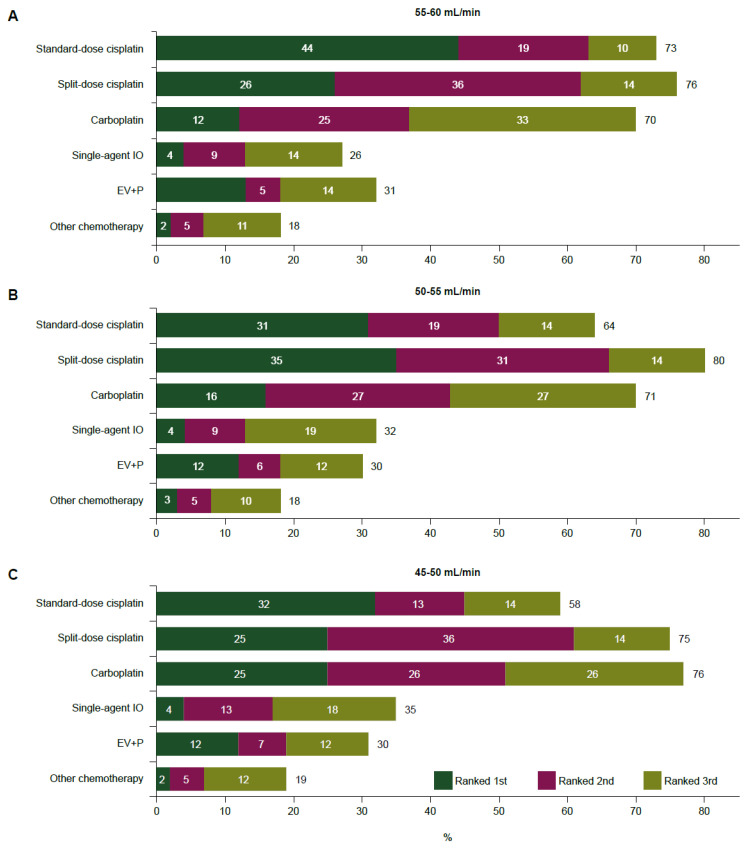
Preferred regimens for otherwise fit patients with la/mUC if their creatinine clearance is (**A**) 55–60 mL/min, (**B**) 50–55 mL/min, or (**C**) 45–50 mL/min (N = 791). Graphs show the percentage of physicians who selected the respective regimen as first, second, or third choice based on regimens available in their clinical practice. EV+P, enfortumab vedotin + pembrolizumab; IO, immunotherapy; la/mUC, locally advanced or metastatic urothelial cancer.

Physicians were asked to rank la/mUC 1L treatments in otherwise fit patients with borderline kidney function according to what regimens were available in their clinical practice (Figure 3). Standard-dose cisplatin was the most common first choice (44%) for patients with a CrCl of 55–60 mL/min, and the percentage of respondents who ranked standard-dose cisplatin in their top three regimens decreased with lower CrCl. Split-dose cisplatin was the first choice for ≥25% of respondents for patients in any queried CrCl range and was the preferred option for patients with a CrCl of 50–55 mL/min (ranked first by 35%; ranked in the top three by 80%). The use of carboplatin consistently increased with lower CrCl, as did the use of single-agent ICIs, which were rarely the first choice. A modest proportion of respondents (12%) selected enfortumab vedotin + pembrolizumab (EV+P) as their first choice of therapy irrespective of CrCl range; 30% of respondents included EV+P among their top three preferred regimens regardless of CrCl range. The ranking of la/mUC 1L treatments in otherwise fit patients with borderline kidney function by geographical region is shown in Supplemental Table S9. Preferred regimens for la/mUC patients with creatinine clearance of 55–60 mL/min varied significantly by region for standard-dose cisplatin (*p* = 0.039), split-dose cisplatin (*p* = 0.007), single-agent immunotherapy (*p* = 0.027), and EV+P (*p* = 0.023). For clearance of 50–55 mL/min, only EV+P preferences differed (*p* = 0.001), whereas for 45–50 mL/min, differences were observed for carboplatin (*p* = 0.008) and EV+P (*p* < 0.001).

Physicians were asked how, based on their previous clinical experience, split-dose cisplatin compared with standard-dose cisplatin and carboplatin in terms of efficacy and toxicity in otherwise fit patients with la/mUC who had a CrCl of 45–55 mL/min (Supplemental Figure S1). The effectiveness of split-dose vs standard-dose cisplatin was rated as a little better or much better by 42% of respondents, whereas 36% saw no difference and 12% saw worse effectiveness with the split regimen. More than 50% of physicians favored split-dose cisplatin (“a little better” or “much better”) in all remaining comparisons; 70% of respondents specifically highlighted the tolerability of split-dose vs. standard-dose cisplatin. Lastly, 78% of respondents agreed that split-dose cisplatin was a reasonable treatment option for otherwise fit patients with la/mUC who had a CrCl of 45–55 mL/min (Supplemental Table S10).

## 4. Discussion

To our knowledge, this is the first international survey on the use of split-dose cisplatin in UC. We found that a large proportion of surveyed physicians currently use split-dose cisplatin in their treatment of UC in the neoadjuvant, adjuvant, and la/mUC disease settings.

Consistent with expectations, the most-used split-dose cisplatin regimen for patients with la/mUC across countries was split-dose GC with a cisplatin dose of 35 mg/m^2^ administered on days 1 and 8 of a 21-day cycle, which was consistent for all geographic regions [13,14,19,20,21]. This regimen has several advantages, including administration of the full total dose of cisplatin (70 mg/m^2^), an opportunity for healthcare providers to carefully monitor the tolerability of cisplatin on days 1 and 8, and similar antiemetic medications on days 1 and 8, which is much simpler for patients to follow. Overall, more than 80% of physicians reported routinely using avelumab 1L maintenance following split-dose cisplatin in the absence of disease progression. This indicates significant uptake of this treatment, which has been shown to prolong survival and delay disease progression in la/mUC [7]. Our analysis indicates that physicians with more years of experience and higher patient loads are more likely to use split-dose cisplatin. More practical experience may increase the willingness to use a split-dose cisplatin regimen, for which clear guidelines are currently lacking.

Several studies have shown that split-dose cisplatin may be a viable option when standard-dose cisplatin cannot be administered, and it does not compromise treatment effectiveness [12,13,14,15,16,22,23]. In a retrospective chart review by Schlack et al., GC, split-dose GC, and GCa resulted in similar outcomes despite differing baseline patient characteristics [13]. In a systemic literature review and network meta-analysis by O’Dwyer et al., split-dose GC demonstrated comparable effectiveness to other regimens, with a higher objective response rate compared with GCa and a similar overall survival rate compared with GC and GCa [12].

In our survey, renal function and ECOG PS were the most frequently considered characteristics when deciding on which platinum-based regimen to use. This finding is consistent with prior surveys asking about platinum eligibility in the la/mUC setting [24,25]. The original definition of cisplatin ineligibility by Galsky et al. included ECOG PS ≥2, CrCl ≤60 mL/min, hearing loss grade ≥2, PN grade ≥2, and/or NYHA class ≥III [10]. The ESMO guideline furthermore states that GC can be considered for otherwise fit patients without comorbidities, a good ECOG PS (0–1), and a CrCl between 50 and 60 mL/min [4], in line with the threshold most commonly chosen in a survey by Galsky et al. of 50 mL/min [26]. A more recent survey by Gupta et al. defined criteria that preclude the use of any platinum-based chemotherapy: ECOG PS ≥3, CrCl <30 mL/min, PN grade ≥2, and NYHA Class ≥III; these have been adopted by the EAU 2024 guideline [2,3,25]. In general, responses in our survey indicate that in clinical practice, split-dose cisplatin is administered to patients with a wider range of CrCl values, ECOG PS, and comorbidities than previously suggested for standard-dose cisplatin. Above a CrCl threshold of 40 mL/min, most physicians (64%) were comfortable prescribing split-dose cisplatin to an otherwise fit patient. For patients with ECOG PS of 2, 70% of respondents would consider prescribing split-dose cisplatin to patients with a CrCl of ≥50 mL/min. The proportion of physicians who would consider administering split-dose cisplatin in patients with ECOG PS of 3, or grade 3 and 4 comorbidities, was slightly higher than expected based on previously reported thresholds for prescribing standard-dose cisplatin. Notably, results indicated that, under specific circumstances, physicians were willing to prescribe cisplatin-based regimens to patients with a higher comorbidity burden under specific circumstances, such as higher CrCl levels. Finally, based on our results, age seemed to play an important role when platinum-based chemotherapy was prescribed, despite not being included in the Galsky criteria. This important finding has also been shown by other studies [27]. Indeed, older patients may benefit from a geriatric assessment to confirm whether or not they are eligible to receive chemotherapy, rather than being categorically excluded based on age alone. Several patient groups recommend routine comprehensive geriatric assessments in older patients, although the availability of resources could be challenging [28].

Platinum-based chemotherapy has been the standard of care for 1L treatment of la/mUC for more than four decades, resulting in a wealth of evidence and practical experience [29]. Cisplatin, when combined with an ICI, is generally considered to have greater activity and immunogenicity than carboplatin [30,31]. At the time of data collection, (first quarter of 2024), split-dose and standard-dose GC represented the preferred treatment choices for otherwise fit patients with borderline kidney function (45–60 mL/min), as shown by the results of this survey. However, a recent phase 3 trial in la/mUC showed superior efficacy with EV+P vs. platinum-based chemotherapy [32]. Consequently, EV+P is recognized as the new standard of care for 1L advanced UC, as indicated by recently updated guidelines [3,5,6]. Although guidelines favor EV+P over platinum-based chemotherapy regardless of platinum eligibility [3,5], access may be limited in some countries or healthcare settings owing to economic constraints, reimbursement policies, and availability of this newer regimen. This limited accessibility, combined with the timing of data collection relative to the publication of these new guidelines, likely explains the low first-choice preference for EV+P in our survey. The preference for EV+P as 1L treatment in patients with la/mUC with borderline kidney function was significantly higher among respondents from North America (~25%) compared with other geographic regions. This disparity highlights regional differences, likely influenced by variations in access, availability, and treatment barriers. Similar accessibility and economic challenges may also apply to another emerging treatment option: 1L nivolumab + GC. As such, platinum-based chemotherapy alone may remain the 1L approach in la/mUC in some jurisdictions, particularly in low- and middle-income countries [33,34,35]. In the 1L setting, nivolumab + GC has shown efficacy benefits compared with GC alone [8]. If split-dose cisplatin can be given in this regimen, it may enable more patients to benefit from nivolumab + GC, who would otherwise be excluded because they are cisplatin-ineligible per Galsky criteria. Defining clear thresholds for the use of split-dose GC eligibility may also enable more patients to be enrolled in clinical trials evaluating cisplatin-based combinations, thereby enhancing the generalizability of study findings. The recently reported NIAGARA phase 3 trial, which examined neoadjuvant GC ± durvalumab and allowed patients with a CrCl of 40–60 mL/min to enroll with the option of receiving a split-dose GC regimen, provides relevant insights into this approach [36]. Notably, 19% of patients in each treatment arm had a CrCl within this range and received split-dose cisplatin-based chemotherapy. This demonstrates the feasibility of patients with renal impairment receiving an alternative cisplatin-based regimen within the context of a clinical trial, highlighting the potential to broaden eligibility criteria and improve generalizability.

Our survey results furthermore suggested potential uptake of split-dose cisplatin in the second-line setting and beyond, which is important given the changes in the 1L setting. Overall, cisplatin is predicted to remain a relatively inexpensive and critically important treatment option for patients with la/mUC in many settings, despite recent advances in the treatment landscape.

Despite the critical role of cisplatin, current guidelines offer limited advice on split-dose cisplatin usage. Our survey confirmed that the ESMO, NCCN, and EAU guidelines are referred to most often by physicians who use systemic anticancer therapy to treat UC. A recent randomized trial that evaluated the safety of split-dose cisplatin with regard to renal toxicity is cited by the ESMO guidelines with no conclusion on its role [4,21]. The NCCN guideline suggests consideration of split-dose cisplatin administration in patients with borderline renal dysfunction and cites a multicenter study by Coleman et al. that supports the use of split-dose GC for upper-tract UC in the neoadjuvant setting [6,37]. Although the current EAU guidelines reference a few studies of split-dose cisplatin in the neoadjuvant [38] and metastatic settings [19,20], these were conducted in 2012 and earlier, without further guidance on the use of this regimen in clinical practice [2,3]. Overall, guidance on the use of split-dose cisplatin in these guidelines is currently sparse, heterogeneous, and inconclusive.

In the muscle-invasive bladder cancer setting, Jiang et al. proposed a multidisciplinary approach to assess cisplatin eligibility, rather than relying on defined CrCl thresholds, to reduce inappropriate exclusion of patients from cisplatin-based chemotherapy [17]. Considering the heterogeneity of thresholds and eligibility criteria chosen by physicians in this survey and the continued role cisplatin will play in the care of patients with UC, clearer guidance on optimal use is urgently needed. Such guidance will help give patients with UC greater access to the activity of cisplatin-based treatments in routine clinical practice and potentially in clinical trials.

### Strengths and Limitations

Strengths of this primary data collection study include the large international sample, a pilot survey that ensured understandability and relevance, translations by medical linguists, mindful phrasing of survey invitations, screening questions to avoid faulty self-selection and bias toward split-dose cisplatin users, and several measures to ensure data quality. Applied quotas increased the representativeness of the study sample to reflect clinical practice.

Several limitations need to be highlighted. Although the applied quotas were intended to increase representativeness, this may have introduced artificial constraints on participant inclusion and restricted the natural variability of physician distribution. A higher proportion of physicians in Spain and Italy were excluded because of the practice-location quota, suggesting that certain types of practice locations may be more prevalent in these countries. Additionally, self-selection and nonresponse bias cannot be ruled out. Voluntary participation may have introduced self-selection bias, possibly overrepresenting physicians more engaged in UC treatment or using split-dose cisplatin, which could skew the results. Nonresponse bias is also possible because excluded or nonparticipating physicians may differ from respondents. This may limit the generalizability of findings compared with the broader population of healthcare providers managing patients with la/mUC. Because the answers were self-reported, the potential exists for response bias, whereby answers may reflect perceived best practices rather than actual behavior. Furthermore, questions about thresholds and eligibility criteria were asked separately for each characteristic in an otherwise fit patient; because the decision to treat a patient with split-dose cisplatin in a real-world setting often depends on a combination of factors, this aspect needs to be kept in mind when interpreting the results.

While the use of a convenience sample may limit the generalizability of the findings, our study provides valuable insights into the treatment decision-making process for split-dose cisplatin in UC and la/mUC across 10 countries.

## 5. Conclusions

Split-dose cisplatin is used by a large proportion of physicians in current day-to-day clinical care of patients with UC and la/mUC around the world, and it is a viable alternative in cases when standard-dose cisplatin is unsuitable. This survey showed that the most common split-dose regimen was cisplatin 35 mg/m^2^ in combination with gemcitabine given on days 1 and 8 of a 21-day cycle; >80% of physicians subsequently used avelumab maintenance.

Responses to our survey indicated that in clinical practice, split-dose cisplatin is administered to patients with a wider range of CrCl levels and comorbidities than previously suggested for standard-dose cisplatin. The heterogeneity of thresholds and eligibility criteria chosen by physicians in this survey emphasize the need for consensus guidelines on the optimal use of split-dose cisplatin in patients unfit for standard-dose cisplatin because platinum-based chemotherapy will continue to play a role in 1L and later lines of treatment. Clear guidance is especially needed to reduce the misclassification of patients as cisplatin-ineligible who are suitable to receive split-dose cisplatin, potentially allowing greater access to cisplatin in the clinical trial setting and improving outcomes in clinical practice.

## Figures and Tables

**Table 1:** Physician demographics and characteristics by geographic region.

	Total	Europe ^a^	North America ^b^	Brazil	India	Australia	*p* Value
	N = 791	n = 375	n = 184	n = 100	n = 107	n = 25	
**Age, years**							<0.001
Mean (SD)	43.2 (9.3)	44.4 (9.1)	45.0 (10.7)	39.2 (6.7)	39.7 (7.4)	43.5 (9.4)	
Median (IQR)	41 (36–50)	44 (37–51)	45 (38–53)	38 (35–42)	39 (35–42)	40 (35–50)	
**Years in clinical practice ^c^**							<0.001
Mean (SD)	13.2 (7.3)	14.1 (6.9)	14.7 (7.8)	9.8 (5.7)	10.8 (6.9)	10.9 (7.9)	
Median (IQR)	12 (7–18)	14 (8–19)	14 (8–20)	8 (6–12)	9 (7–14)	8 (4–16)	
**Gender, n (%)**							0.001
Female	210 (26.5)	110 (29.3)	32 (17.4)	46 (46.0)	16 (15.0)	6 (24.0)	
Male	578 (73.1)	264 (70.4)	151 (82.1)	54 (54.0)	91 (85.0)	18 (72.0)	
Other	3 (0.4)	1 (0.3)	1 (0.5)	0 (0)	0 (0)	1 (4.0)	
**Medical specialty, n (%)**							<0.001
Medical oncologist	725 (91.7)	325 (86.7)	184 (100)	100 (100)	91 (85.0)	25 (100)	
Urologist	66 (8.3)	50 (13.3)	0 (0)	0 (0)	16 (15.0)	0 (0)	
**Practice setting, n (%)**							<0.001
Private	271 (34.3)	30 (8.0)	61 (33.2)	88 (88.0)	90 (84.1)	2 (8.0)	
Public	436 (55.1)	314 (83.7)	92 (50.0)	4 (4.0)	13 (12.1)	13 (52.0)	
I spend equal time in either setting	84 (10.6)	31 (8.3)	31 (16.8)	8 (8.0)	4 (3.7)	10 (40.0)	
**Principal practice location, n (%)**							<0.001
Academic/teaching hospital or specialized cancer center	465 (58.8)	255 (68.0)	103 (56.0)	44 (44.0)	42 (39.3)	21 (84.0)	
Community/nonteaching hospital	212 (26.8)	89 (23.7)	51 (27.7)	6 (6.0)	64 (59.8)	2 (8.0)	
Office-based practice	114 (14.4)	31 (8.3)	30 (16.3)	50 (50.0)	1 (0.9)	2 (8.0)	
**Hospital/center size,** **n (%)**							<0.001
Large (≥500 beds)	404 (51.1)	235 (62.7)	92 (50.0)	22 (22.0)	40 (37.4)	15 (60.0)	
Medium (100–499 beds)	231 (29.2)	97 (25.9)	56 (30.4)	26 (26.0)	46 (43.0)	6 (24.0)	
Small (<100 beds)	36 (4.6)	7 (1.9)	6 (3.3)	2 (2.0)	19 (17.8)	2 (8.0)	
Don’t know/unsure	6 (0.8)	5 (1.3)	0 (0)	0 (0)	1 (0.9)	0 (0)	
NA (office-based practice)	114 (14.4)	31 (8.3)	30 (16.3)	50 (50.0)	1 (0.9)	2 (8.0)	
**Number of monthly new (first-time) patients with UC**							<0.001
Mean (SD)	24.9 (44.8)	24.9 (44.8)	32.0 (52.8)	27.8 (45.6)	11.7 (15.1)	11.7 (25.8)	
Median (IQR)	10 (5–28)	10 (5–28)	16 (10–35)	12 (5–30)	6 (4–15)	5 (3–10)	
**Number of monthly new (first-time) and follow-up patients with UC**							<0.001
Mean (SD)	42.0 (59.2)	51.6 (66.3)	47.1 (65.8)	23.3 (23.8)	21.4 (34.9)	23.7 (30.6)	
Median (IQR)	25 (12–50)	30 (20–60)	25 (15–50)	15 (10–26)	10 (6–23)	10 (8–20)	
**Number of monthly new (first time) patients with la/mUC treated**							<0.001
Mean (SD)	28.5 (44.5)	35.3 (49.3)	33.4 (52.7)	16.2 (18.1)	10.7 (16.6)	14.8 (17.3)	
Median (IQR)	15 (8–30)	20 (10–40)	18 (10–30)	10 (5–20)	5 (3–10)	8 (4–15)	
**Enrollment of patients with UC in clinical trials, n (%)**							<0.001
Yes	395 (49.9)	211 (56.3)	92 (50.0)	47 (47.0)	26 (24.3)	19 (76.0)	
No	396 (50.1)	164 (43.7)	92 (50.0)	53 (53.0)	81 (75.7)	6 (24.0)	
**Guidelines considered for the treatment of UC, n (%) ^d^**							
EAU	271 (34.3)	184 (49.1)	26 (14.1)	22 (22.0)	33 (30.8)	6 (24.0)	<0.001
ESMO	476 (60.2)	302 (80.5)	45 (24.5)	72 (72.0)	39 (36.4)	18 (72.0)	<0.001
NCCN	490 (61.9)	152 (40.5)	154 (83.7)	80 (80.0)	85 (79.4)	19 (76.0)	<0.001
AUA/SUO	115 (14.5)	52 (13.9)	38 (20.7)	11 (11.0)	11 (10.3)	3 (12.0)	<0.001
ASCO	381 (48.2)	156 (41.6)	110 (59.8)	81 (81.0)	20 (18.7)	14 (56.0)	<0.001
National guideline(s)	143 (18.1)	103 (27.5)	6 (3.3)	26 (26.0)	4 (3.7)	4 (16.0)	0.0665
Other international guideline(s)	6 (0.8)	4 (1.1)	0 (0)	1 (1.0)	1 (0.9)	0 (0)	0.1344
Institutional guideline(s)	100 (12.6)	53 (14.1)	16 (8.7)	14 (14.0)	11 (10.3)	6 (24.0)	0.4008
None ^e^	11 (1.4)	7 (1.9)	2 (1.1)	1 (1.0)	0 (0)	1 (4.0)	0.7491
**Method to assess kidney function in patients with UC being considered for platinum-based** **chemotherapy, n (%) ^d^**							<0.001
Calculated creatinine clearance	680 (86.0)	337 (89.9)	149 (81.0)	90 (90.0)	81 (75.7)	23 (92.0)	
Measured creatinine clearance	196 (24.8)	106 (28.3)	58 (31.5)	20 (20.0)	10 (9.3)	2 (8.0)	
Measured GFR	251 (31.7)	141 (37.6)	75 (40.8)	17 (17.0)	12 (11.2)	6 (24.0)	
Serum creatinine value	338 (42.7)	155 (41.3)	96 (52.2)	40 (40.0)	42 (39.3)	5 (20.0)	
Other	5 (0.6)	2 (0.5)	1 (0.5)	0 (0)	2 (1.9)	0 (0)	
**Formula used to calculate creatinine clearance, n (%) ^f^**	**n = 680**	**n = 337**	**n = 149**	**n = 90**	**n = 81**	**n = 23**	
Cockcroft-Gault equation	462 (67.9)	202 (59.9)	108 (72.5)	62 (68.9)	72 (88.9)	18 (78.3)	0.002
Modification of Diet in Renal Disease equation	82 (12.1)	56 (16.6)	9 (6.0)	11 (12.2)	5 (6.2)	1 (4.3)	
Chronic Kidney Disease Epidemiology Collaboration equation	99 (14.6)	52 (15.4)	25 (16.8)	16 (17.8)	2 (2.5)	4 (17.4)	
Jelliffe equation	4 (0.6)	3 (0.9)	0 (0)	0 (0)	1 (1.2)	0 (0)	
Wright equation	6 (0.9)	4 (1.2)	2 (1.3)	0 (0)	0 (0)	0 (0)	
Unsure	24 (3.5)	19 (5.6)	3 (2.0)	1 (1.1)	1 (1.2)	0 (0)	
Other	3 (0.4)	1 (0.3)	2 (1.3)	0 (0)	0 (0)	0 (0)	
**Prescribes split-dose cisplatin (any setting), n (%)**	670 (84.7)	334 (89.1)	168 (91.3)	67 (67.0)	82 (76.6)	19 (76.0)	<0.001
	**n = 670 ^g^**	**n = 334**	**n = 168**	**n = 67**	**n = 82**	**n = 19**	
**Percentage of patients treated with split-dose** **cisplatin in the neoadjuvant setting**							<0.001
Mean (SD)	40.9 (26.3)	34.7 (22.5)	39.0 (22.4)	70.0 (25.5)	49.0 (30.1)	27.8 (28.3)	
Median (IQR)	34 (20–56)	30 (20–50)	35 (20–50)	70 (50–98)	50 (20–75)	20 (10–32)	
**Percentage of patients treated with split-dose** **cisplatin in the adjuvant setting**							<0.001
Mean (SD)	39.5 (26.0)	34.4 (22.4)	37.8 (22.7)	64.4 (27.8)	47.3 (31.1)	21.7 (21.5)	
Median (IQR)	35 (20–54)	30 (20–50)	35 (20–50)	65 (50–83)	50 (20–75)	20 (8–20)	
**Percentage of patients treated with split-dose** **cisplatin in the advanced setting**							<0.001
Mean (SD)	43.0 (26.8)	36.8 (22.8)	40.1 (23.8)	70.2 (27.9)	54.4 (30.7)	33.3 (22.6)	
Median (IQR)	40 (20–60)	30 (20–50)	35 (24–51)	80 (50–100)	50 (30–80)	33 (15–48)	

ASCO, American Society of Clinical Oncology; AUA, American Urological Association; EAU, European Association of Urology; ESMO, European Society for Medical Oncology; GC, gemcitabine + cisplatin; GFR, glomerular filtration rate; IQR, interquartile range; la/mUC, locally advanced or metastatic urothelial cancer; N/A, not applicable; NCCN, National Comprehensive Cancer Network; SD, standard deviation; SUO, Society of Urologic Oncology; UC, urothelial cancer. ^a^ Europe includes respondents from France, Germany, Italy, Spain, and the UK. ^b^ North America includes respondents from USA and Canada. ^c^ Years in clinical practice since the end of medical specialist training. ^d^ Multiple answers possible. ^e^ “I do not consider specific guidelines for the treatment of [patients with UC]”. ^f^ Assessed only in respondents who calculated creatinine clearance. ^g^ Questions asked to physicians who indicated they generally prescribe split-dose cisplatin to patients with UC.

**Table 2:** Details on split-dose cisplatin use in la/mUC.

	Total N = 660	USA n = 135	India n = 81	Brazil n = 67	Germany n = 67	France n = 70	UK n = 60	Italy n = 69	Spain n = 64	Canada n = 29	Australia n = 18
**Within the unresectable locally advanced or metastatic setting, in which line(s) of treatment do you consider using split-dose cisplatin? (multiple answers possible)**											
1L	548 (83.0)	102 (75.6)	62 (76.5)	64 (95.5)	55 (82.1)	62 (88.6)	51 (85.0)	61 (88.4)	53 (82.8)	23 (79.3)	15 (83.3)
2L	340 (51.5)	87 (64.4)	26 (32.1)	31 (46.3)	40 (59.7)	29 (41.4)	42 (70.0)	30 (43.5)	32 (50.0)	18 (62.1)	5 (27.8)
3L	168 (25.5)	51 (37.8)	10 (12.3)	14 (20.9)	21 (31.3)	15 (21.4)	20 (33.3)	14 (20.3)	15 (23.4)	4 (13.8)	4 (22.2)
**Which split-dose cisplatin regimens do you use in patients with la/mUC? (top five regimens; multiple answers possible)**											
GC 35 mg/m^2^, days 1 + 8 of 21-day cycles	378 (57.3)	75 (55.6)	39 (48.1)	48 (71.6)	36 (53.7)	36 (51.4)	36 (60.0)	42 (60.9)	35 (54.7)	17 (58.6)	14 (77.8)
GC 25 mg/m^2^, days 1 + 8 of 21-day cycles	86 (13.0)	15 (11.1)	14 (17.3)	9 (13.4)	5 (7.5)	11 (15.7)	9 (15.0)	10 (14.5)	11 (17.2)	2 (6.9)	0
GC 35 mg/m^2^, days 1 + 8 of 28-day cycles	36 (5.5)	7 (5.2)	6 (7.4)	0	8 (11.9)	2 (2.9)	3 (5.0)	4 (5.8)	2 (3.1)	3 (10.3)	1 (5.6)
GC 35 mg/m^2^, days 1 + 2 of 21-day cycles	25 (3.8)	4 (3.0)	6 (7.4)	3 (4.5)	3 (4.5)	4 (5.7)	1 (1.7)	0	1 (1.6)	1 (3.4)	2 (11.1)
GC 35 mg/m^2^, days 1 + 15 of 21-day cycles	20 (3.0)	6 (4.4)	2 (2.5)	0	1 (1.5)	2 (2.9)	2 (3.3)	2 (2.9)	5 (7.8)	0	0
**In patients with la/mUC, do you routinely use avelumab as first-line maintenance if there is no disease progression during or after split-dose cisplatin-based chemotherapy?**											
Yes	531 (80.5)	111 (82.2)	23 (28.4)	62 (92.5)	53 (79.1)	66 (94.3)	49 (81.7)	67 (97.1)	60 (93.8)	22 (75.9)	18 (100)
No	129 (19.5)	24 (17.8)	58 (71.6)	5 (7.5)	14 (20.9)	4 (5.7)	11 (18.3)	2 (2.9)	4 (6.3)	7 (24.1)	0

n (%) is shown. 1L, first line; 2L, second line; 3L, third line; GC, gemcitabine + cisplatin; la/mUC, locally advanced or metastatic urothelial cancer.

**Table 3:** Predictors of prescribing split-dose cisplatin to patients with UC across countries, multivariable logistic regression model.

Covariate	OR (95% CI)	*p* Value
**Model excluding geographic region**		
Specialty: oncologist	1 (ref)	
Specialty: urologist	0.45 (0.24–0.88)	0.016
Practice years (per decade)	1.58 (1.18–2.16)	0.003
Practice setting: private	1 (ref)	
Practice setting: public	1.94 (1.27–2.97)	0.002
Practice setting: I spend equal time in either setting	2.58 (1.21–6.17)	0.021
Total number of patients with UC per month (per 10 patients)	1.06 (1.01–1.14)	0.045
**Model including geographic region**		
Specialty: oncologist	1 (ref)	
Specialty: urologist	0.38 (0.19–0.77)	0.006
Practice years (per decade)	1.37 (1.01–1.90)	0.047
Practice setting: private	1 (ref)	
Practice setting: public	1.12 (0.60–2.09)	0.713
Practice setting: I spend equal time in either setting	2.04 (0.88–5.24)	0.113
Total number of patients with UC per month (per 10 patients)	1.05 (1.00–1.12)	0.093
Region: Europe ^a^	1 (ref)	
Region: North America ^b^	1.11 (0.59–2.20)	0.748
Region: Brazil	0.30 (0.14–0.64)	0.002
Region: India	0.58 (0.27–1.22)	0.149
Region: Australia	0.35 (0.13–1.06)	0.051

OR, odds ratio; UC, urothelial cancer. ^a^ Europe includes respondents from France, Germany, Italy, Spain, and the UK. ^b^ North America includes respondents from USA and Canada.

## Data Availability

Any requests for data by qualified scientific and medical researchers for legitimate research purposes will be subject to Merck’s Data Sharing Policy. All requests should be submitted in writing to Merck’s data sharing portal (https://www.merckgroup.com/en/research/our-approach-to-research-and-development/healthcare/clinical-trials/commitment-responsible-data-sharing.html, accessed on 28 January 2025). When Merck has a co-research, co-development, or co-marketing or co-promotion agreement, or when the product has been out-licensed, the responsibility for disclosure might be dependent on the agreement between parties. Under these circumstances, Merck will endeavor to gain agreement to share data in response to requests.

## Supplementary Materials

The following supporting information can be downloaded at: https://www.mdpi.com/article/10.3390/cancers17030509/s1, Table S1. Final English questionnaire. Table S2. Participant selection and exclusion reasons. Table S3. Physician demographics and characteristics by country. Table S4. Details on split-dose cisplatin use in la/mUC by geographic region. Table S5. Reasons for not using split-dose cisplatin. Table S6. Predictors of prescribing split-dose cisplatin to patients with UC across countries: univariable analysis. Table S7. Predictors of prescribing split-dose cisplatin to patients with UC by geographic region: multivariable logistic regression model. Table S8. Consideration of characteristics in physician’s decision to prescribe split-dose cisplatin by geographical region. Table S9. Preferred regimens for otherwise fit patients with la/mUC with borderline kidney function based on creatinine clearance, by geographical region. Table S10. Split-dose cisplatin as a treatment option for otherwise fit patients with la/mUC and borderline kidney function. Figure S1. Comparison of split-dose cisplatin to other regimens in otherwise fit patients with locally advanced or metastatic urothelial cancer and a creatinine clearance of 45 to 55 mL/min.
