# Supplementary material for: Split-Dose Cisplatin Use, Eligibility Criteria, and Drivers for Treatment Choice in Patients with Locally Advanced or Metastatic Urothelial Carcinoma: Results of a Large International Physician Survey
Source: Cancers (Basel). 2025 Feb 3;17(3):509. doi: 10.3390/cancers17030509 (PMC11816825; doi:10.3390/cancers17030509)

## SUPPLEMENTARY MATERIAL

Split-dose cisplatin use, eligibility criteria, and drivers for treatment choice in patients with locally advanced or metastatic urothelial carcinoma: results of a large international physician survey (O'Dwyer R. et al.)

**Supplemental Table S1. Final English questionnaire.**

<table>
<tr><th>No.</th><th>Item</th></tr>
<tr><td colspan="2">SCREENER</td></tr>
<tr><td>S1</td><td>Please confirm the country in which you are practicing.<br>O (1) USA<br>O (2) India<br>O (3) Brazil<br>O (4) Germany<br>O (5) France<br>O (6) United Kingdom<br>O (7) Italy<br>O (8) Spain<br>O (9) Canada<br>O (10) Australia<br>O (99) Other<br>[Terminate immediately if "Other".]</td></tr>
<tr><td>S1b</td><td>[Only display if answer to item S1 is "USA".]<br>Are you board-certified or board-eligible to practice in the USA?<br>O (1) Yes<br>O (0) No<br>[Terminate immediately if "No".]</td></tr>
<tr><td>S2</td><td>Please confirm your medical specialty.<br>☐ (1) Medical oncologist<br>☐ (2) Urologist<br>☐ (3) Hematologist<br>☐ (4) Gynecologist<br>☐ (99) Other<br>[Qualitative pilot: Terminate immediately if "Medical oncologist" is not selected. If answer to S1 is "Germany", terminate immediately if neither "Medical oncologist" nor "Urologist" are selected.<br>Quantitative study: Terminate immediately if neither "Medical oncologist" nor "Urologist" are selected.<br>See study protocol for implementation of quotas per country.] [If answer to S1 is "USA", terminate immediately if not "Medical oncologist"]</td></tr>
<tr><td>S3</td><td>Please confirm how many years you have been in practice since the end of your medical specialist training.<br>|__|__| years [Possible range: 0-70]<br>[Qualitative pilot: Terminate immediately if <10.<br>Quantitative study: Terminate immediately if <2.]</td></tr>
<tr><td>S4</td><td>Please confirm how many patients you have treated within the past 12 months for the following conditions (count each patient only once):<br>Breast cancer: |__|__|__|__| patients [Possible range: 0-1000]<br>Urothelial cancer: |__|__|__|__| patients [Possible range: 0-1000]<br>Prostate cancer: |__|__|__|__| patients [Possible range: 0-1000]<br>[Qualitative pilot: Terminate immediately if Urothelial cancer < 20.<br>Quantitative study: Terminate immediately if Urothelial cancer < 10.]</td></tr>
<tr><td>S5</td><td>Out of the XX [Insert number indicated for Urothelial cancer from item S4] patients with urothelial cancer you have treated in the past 12 months, how many had unresectable locally advanced or metastatic urothelial cancer?<br>|__|__|__|__| patients [Possible range: 0-S4_02]</td></tr>
</table>

| No. | Item |
| --- | --- |
| | *[Qualitative pilot: Terminate immediately if < 10.<br>Quantitative study: Terminate immediately if < 5.]* |
| S6 | At your primary practice location, what is your level of decision making with regard to prescribing and overseeing systemic anticancer therapy?<br>O (1) No responsibility<br>O (2) Little responsibility<br>O (3) Large responsibility<br>O (4) Full responsibility<br>*[Terminate immediately if "No responsibility" or "Little responsibility".]* |
| S7a | Please confirm the setting (public/private) in which you spend the most hours per week.<br>O (1) Public setting<br>O (2) Private setting<br>O (3) I spend equal time in either setting |
| S7 | Please confirm which of the following categories BEST describes your principal practice location (i.e., the practice location where you spend the most hours per week).<br><br>O (1) Academic/teaching hospital or specialized cancer center / UK, India: Academic/teaching hospital or specialized (tertiary) cancer center / Italy: Academic/teaching hospital or specialized cancer center/IRCCS<br>O (2) Community/nonteaching hospital / UK: Community/nonteaching hospital/district general hospital<br>O (3) Office-based practice<br>*[See study protocol for implementation of quotas.]* |
| S8 | *[Only display in qualitative pilot phase.]*<br>Have you ever prescribed a split-dose cisplatin regimen to one of your patients with unresectable locally advanced or metastatic urothelial cancer? (An example of a split-dose cisplatin regimen is: cisplatin 35mg/m$^2$ and gemcitabine 1000 mg/m$^2$ day 1+8, every 21 days.)<br>O (1) Yes<br>O (0) No<br>*[Terminate immediately if "No".]* |

***QUESTIONNAIRE***

*[Show text below]*

**Please read carefully before starting the survey**:

Thank you for agreeing to take part in this research survey, which is conducted by Cytel Inc., a healthcare consulting agency, on behalf of a pharmaceutical company.

Cisplatin-based chemotherapy is an integral part of the management of urothelial cancer (UC). However, many patients have kidney dysfunction and other comorbidities which often precludes them from receiving cisplatin-based chemotherapy. In patients who may be considered ineligible for standard-dose cisplatin-based chemotherapy, modified regimens such as split-dose cisplatin are sometimes used rather than carboplatin or non-platinum containing regimens.

An example of a split-dose cisplatin regimen is the following:

Cisplatin 35 mg/m$^2$ and gemcitabine 1000 mg/m$^2$ day 1+8, every 21 days

This international survey is intended to collect information on the use of split-dose cisplatin in patients with UC across 10 countries and to help to develop guidelines for the use of such regimens in the future. The survey will take approximately 25 minutes to complete. <u>Please consider completing this survey even if you do not use split-dose cisplatin regimens in your daily practice.</u>

Taking this opportunity to have your voice heard would help us further our research, and your participation would be greatly appreciated.

**Privacy note**

The research will comply with all relevant local and regional laws including GDPR and good data protection practices outside of the EU. Furthermore, the research will be in accordance with the codes of conduct as set out by market research associations including but not limited to EphMRA, ESOMAR and BHBIA within the EU, Insights Association within the US, and MRS globally.

| No. | Item |
|---|---|
| | Any information you provide us with will be treated as confidential; it will be combined with feedback from others like yourself. You will remain anonymous unless you give permission to be identified. Cytel Inc. will receive anonymized data only.<br>Your information will only be used by us for scientific research purposes and will not be passed to any other organization without your permission. Only aggregated study results may be published.<br>You have the right to refuse to answer questions or withdraw at any time. |
| A1 | What is your gender?<br>O (1) Female<br>O (2) Male<br>O (3) Other |
| A2 | What is your age (years) today?<br>O \|__\|__\| years *[Possible range: 18-99]* |
| A3 | *[Display only if answer to S7 is "(1) Academic/teaching hospital or specialized center", "(2) Community/non-teaching hospital]*<br>Is your hospital/center considered a large, medium, or small hospital/center? If you work at more than one hospital, please describe your principal hospital location.<br>O (1) Large (500 or more beds)<br>O (2) Medium (100-499 beds)<br>O (3) Small (Fewer than 100 beds)<br>O (97) Don't know/unsure |
| A4 | Do you currently enroll patients with UC in clinical trials?<br>O (1) Yes<br>O (0) No |
| A6 | On average, during a typical month, how many <u>new</u> (first time) patients do you treat for <u>all stages</u> of UC?<br>O \|__\|__\|__\| patients *Range [1-999]* |
| A5 | On average, during a typical month, how many <u>new</u> (first-time) patients and follow-up patients do you treat for <u>all stages</u> of UC? (Please enter the total of new + follow-up patients.)<br>O \|__\|__\|__\| patients *Range [A5-999]* |
| A7 | On average, during a typical month, how many <u>new</u> (first-time) patients and follow-up patients do you treat for unresectable locally advanced or metastatic UC? (Please enter the total of new + follow-up patients.)<br>O \|__\|__\|__\| patients *Range [1-A6]* |
| | *[Show text below]*<br>This section refers to the management of patients with <u>all disease stages</u> of urothelial cancer (UC). |
| B1 | Which guidelines (if any) do you consider for the treatment of patients with UC?<br>☐ (1) EAU Guideline (European Association of Urology)<br>☐ (2) ESMO Guideline (European Society for Medical Oncology)<br>☐ (3) NCCN Guideline (National Comprehensive Cancer Network)<br>☐ (4) AUA/SUO Guideline (American Urological Association/Society of Urologic Oncology)<br>☐ (8) ASCO Guideline (American Society of Clinical Oncology)<br>☐ (5) [Answer from S1] national guideline(s), please specify: __________________<br>☐ (6) Other international guideline(s), please specify: ___________________<br>☐ (7) Institutional guideline(s) (e.g., guidelines at your hospital/center)<br>☐ (0) I do not consider specific guidelines for the treatment of UC patients *[exclusive]* |
| B2a | How do you routinely assess kidney function in patients with UC being considered for platinum-based chemotherapy? Please select all that apply.<br>☐ (1) Calculated creatinine clearance (by formulae using serum creatinine)<br>☐ (2) Measured creatinine clearance (by 24-hour urine collection)<br>☐ (3) Measured GFR (by serum inulin or radioisotope measurement)<br>☐ (4) Serum creatinine value<br>☐ (99) Other, please specify: __________ |
| B2b | *[Display only if one of the answers to B2a is "calculated creatinine clearance".]*<br>Which formula do you usually use to calculate creatinine clearance?<br>O (1) Cockcroft-Gault equation |

| No. | Item |
|---|---|
| | O (2) Modification of Diet in Renal Disease (MDRD) equation<br>O (3) Chronic Kidney Disease Epidemiology Collaboration (CKD-EPI) equation<br>O (4) Jelliffe equation<br>O (5) Wright equation<br>O (97) Unsure<br>O (99) Other, please specify: ___________ |
| B3 | Do you ever use split-dose cisplatin regimens in patients with UC? An example of a split-dose cisplatin regimens is: cisplatin 35mg/m$^2$ and gemcitabine 1000mg/m$^2$ day 1+8, every 21 days.<br>O (1) Yes<br>O (0) No |
| B4a | *[Display only if answer to B3 is "Yes".]*<br>Out of all patients with muscle-invasive bladder cancer that you treat in the neoadjuvant setting, approximately what percentage do you treat with split-dose cisplatin?<br>O \|__\|__\|% |
| B4b | *[Display only if answer to B3 is "Yes".]*<br>Out of all patients with muscle-invasive bladder cancer that you treat in the adjuvant setting, approximately what percentage do you treat with split-dose cisplatin?<br>O \|__\|__\|% |
| B4c | *[Display only if answer to B3 is "Yes".]*<br>Out of all patients with unresectable locally advanced or metastatic UC that you treat, approximately what percentage do you treat with split-dose cisplatin?<br>O \|__\|__\|% |
| B5a | *[Display only if answer to B3 is "Yes" and answer to B4c is not "0".]*<br>Within the unresectable locally advanced or metastatic setting, in which line(s) of treatment do you consider using split-dose cisplatin? Please select all that apply.<br>☐ (1) 1L<br>☐ (2) 2L<br>☐ (3) 3L+ |
| B5b | *[Display only if answer to B3 is "Yes" and answer to B4c is not "0".] [Once an additional regimen is selected, all fields corresponding to that regimen should be mandatory (mandatory to select an option for each field)]*<br>Which split-dose cisplatin regimens do you use in patients with metastatic UC? Please enter the regimen you most commonly use first and enter all that apply.<br>(see table below)<br>☐ Add another split-dose regimen *[If selected, another row with regimen selection options will appear. Max. number of possible regimens: 3.]*<br>*MVAC – methotrexate, vinblastine, doxorubicin and cisplatin* |
| B5c | *[Display only if answer to B3 is "Yes" and answer to B4c is not "0".]*<br>In patients with unresectable locally advanced or metastatic UC, do you routinely use avelumab as first-line maintenance if there is no disease progression during or after split-dose cisplatin-based chemotherapy?<br>O (1) Yes<br>O (0) No |
| B6 | *[Display only if answer to B3 is "No".]*<br>What is your rationale for not using split-dose cisplatin? Please select all that apply.<br>☐ (1) Lack of evidence for efficacy<br>☐ (2) Clinical experience of poor efficacy<br>☐ (3) Concerns regarding toxicity in patients with poor renal function<br>☐ (4) Not part of institutional treatment protocol/guidance<br>☐ (5) Not familiar with this regimen |

B5b table:

| | **Cisplatin dosage** | **Cycle length** | **Cisplatin schedule** | **Combination agent** |
|---|---|---|---|---|
| Split-dose cisplatin regimen 1: | *Drop-down:*<br>(35) *35 mg/m$^2$*<br>(25) *25 mg/m$^2$*<br>(99) *Other:* ___ | *Drop-down:*<br>(21) *21 days*<br>(28) *28 days*<br>(99) *Other:* ___ | *Drop-down:*<br>(8) *Days 1+8*<br>(2) *Days 1+2*<br>(15) *Days 1+15*<br>(99) *Other:* ___ | *Drop-down:*<br>(1) *Gemcitabine/cisplatin*<br>(2) *MVAC*<br>(3) *None (cisplatin monotherapy)*<br>(99) *Other:* ___ |

| No. | Item |
|---|---|
| | ☐ (99) Other, please specify: _________ |
| | *[Show text below]*<br>This section specifically refers to the management of patients with unresectable locally advanced or metastatic urothelial cancer. For simplicity, patients with unresectable locally advanced disease or metastatic disease are referred to as patients with mUC. |
| C2a | Do you consider ECOG PS when deciding on the type of platinum-based chemotherapy in a patient with mUC?<br>O (1) Yes<br>O (0) No<br>*ECOG PS – Eastern Cooperative Oncology Group performance status* |
| C2b | *[Display only if the answer to C2a is "Yes" and answer to B3 is "Yes" and answer to B4c is not "0".]*<br>If you were to treat a patient with mUC, for which of the following ECOG PS scores would you prescribe split-dose cisplatin rather than standard-dose cisplatin? Please select all that apply.<br>☐ ECOG PS 0<br>☐ ECOG PS 1<br>☐ ECOG PS 2<br>☐ ECOG PS 3<br>☐ I would not prescribe split-dose cisplatin in any of these scenarios *[exclusive]*<br>*ECOG PS – Eastern Cooperative Oncology Group Performance Status* |
| C3a | Do you consider kidney function when deciding on the type of platinum-based chemotherapy in a patient with mUC with ECOG PS 0-1 and no other comorbidities affecting treatment decision?<br>O (1) Yes<br>O (0) No |
| C3b | *[Display only if answer to C3a is "Yes" and answer to B3 is "Yes" and answer to B4c is not "0".]*<br>If you were to treat a patient with mUC with ECOG PS 0-1 and no other comorbidities affecting treatment decision, in which range of creatinine clearance would you prescribe split-dose cisplatin rather than standard-dose cisplatin? |

| Lower threshold: | | Upper threshold: |
|---|---|---|
| *Dropdown:*<br>O (25) ≥25 mL/min<br>O (30) ≥30 mL/min<br>O (35) ≥35 mL/min<br>O (40) ≥40 mL/min<br>O (45) ≥45 mL/min<br>O (50) ≥50 mL/min<br>O (55) ≥55 mL/min<br>O (60) ≥60 mL/min<br>O (99) Other: ≥__________ mL/min<br>O (0) No lower creatinine clearance threshold | – | *Dropdown:*<br>O (25) ≤25 mL/min<br>O (30) ≤30 mL/min<br>O (35) ≤35 mL/min<br>O (40) ≤40 mL/min<br>O (45) ≤45 mL/min<br>O (50) ≤50 mL/min<br>O (55) ≤55 mL/min<br>O (60) ≤60 mL/min<br>O (65) ≤65 mL/min<br>O (99) Other: ≤__________ mL/min<br>O (0) No upper creatinine clearance threshold |

*[Upper threshold must be higher than lower threshold.]*

| No. | Item |
|---|---|
| C3c | *[Display only if answer to C3a is "Yes" and answer to B3 is "Yes" and answer to B4c is not "0".]*<br>If you were to treat a patient with mUC with ECOG PS 2, in which range of creatinine clearance would you prescribe split-dose cisplatin rather than standard-dose cisplatin? |

| Lower threshold: | | Upper threshold: |
|---|---|---|
| *Dropdown:*<br>O (30) ≥30 mL/min<br>O (35) ≥35 mL/min<br>O (40) ≥40 mL/min<br>O (45) ≥45 mL/min | – | *Dropdown:*<br>O (35) ≤35 mL/min<br>O (40) ≤40 mL/min<br>O (45) ≤45 mL/min<br>O (50) ≤50 mL/min |

| No. | Item |
|---|---|
| | O (50) ≥50 mL/min<br>O (55) ≥55 mL/min<br>O (60) ≥60 mL/min<br>O (99) Other: ≥__________ mL/min<br>O (0) No lower creatinine clearance threshold<br><br>O (55) ≤55 mL/min<br>O (60) ≤60 mL/min<br>O (65) ≤65 mL/min<br>O (65) ≤70 mL/min<br>O (99) Other: ≤__________ mL/min<br>O (0) No upper creatinine clearance threshold<br>*[Upper threshold must be higher than lower threshold. The lower threshold must be at least as high as lower threshold for C3b. Otherwise error message "The lower threshold for creatinine clearance for a patient with ECOG = 2 should be at least as high as the lower threshold you indicated for a patient with ECOG = 0"]*<br>O I would not prescribe any cisplatin regimen in a patient with ECOG PS 2 *[exclusive]*<br>*["I would not prescribe any cisplatin regimen…" can only be selected if C2b_3 ("ECOG PS 2") is NOT selected. Otherwise, error message "In an earlier question, you indicated that you would use split-dose cisplatin in a patient with ECOG = 2. Please select a creatinine clearance range for such a patient."]* |
| C4a | Do you consider existing heart failure when deciding on the type of platinum-based chemotherapy in a patient with mUC with ECOG PS 0-1 and no other comorbidities affecting treatment decision?<br>O (1) Yes<br>O (0) No |
| C4b | *[Display only if answer to C4a is "Yes" and answer to B3 is "Yes" and answer to B4c is not "0".]*<br>If you were to treat a patient with mUC with ECOG PS 0-1 and no other comorbidities affecting treatment decision, for which of the following functional classes of heart failure would you prescribe split-dose cisplatin rather than standard-dose cisplatin? Please select all that apply.<br>*Please note: If you are not familiar with the New York Heart Association (NYHA) classification, please familiarize yourself with the definitions below and answer accordingly.*<br>• NYHA Class I: no limitation in activity<br>• NYHA Class II: mild symptoms and slight limitation during ordinary activity<br>• NYHA Class III: marked limitation in activity due to symptoms, even during less-than-ordinary activity<br>• NYHA Class IV: severe limitations. Experiences symptoms even while at rest<br><br>☐ NYHA I<br>☐ NYHA II<br>☐ NYHA III<br>☐ NYHA IV<br>☐ I would not prescribe split-dose cisplatin in any of these scenarios *[exclusive]* |
| C5a | Do you consider existing peripheral neuropathy when deciding on the type of platinum-based chemotherapy in a patient with mUC with ECOG PS 0-1 and no other comorbidities affecting treatment decision?<br>O (1) Yes<br>O (0) No |
| C5b | *[Display only if answer to C5a is "Yes" and answer to B3 is "Yes" and answer to B4c is not "0".]*<br>If you were to treat a patient with mUC with ECOG PS 0-1 and no other comorbidities affecting treatment decision, for which of the following peripheral neuropathy (PN) grades would you prescribe split-dose cisplatin rather than standard-dose cisplatin? Please select all that apply.<br>• Grade 1: asymptomatic, loss of deep tendon reflexes<br>• Grade 2: moderate symptoms, limiting instrumental activities of daily living (ADL)<br>• Grade 3: severe symptoms, limiting self-care ADL<br>• Grade 4: Life-threatening consequences; urgent intervention indicated<br><br>☐ PN grade 1<br>☐ PN grade 2<br>☐ PN grade 3<br>☐ PN grade 4<br>☐ I would not prescribe split-dose cisplatin in any of these scenarios *[exclusive]* |

<table>
<tr><th>No.</th><th>Item</th></tr>
<tr><td>C5.1a</td><td>Do you consider <u>audiometric hearing loss</u> when deciding on the type of platinum-based chemotherapy in a patient with mUC with ECOG PS 0-1 and no other comorbidities affecting treatment decision?<br>O (1) Yes<br>O (0) No</td></tr>
<tr><td>C5.1b</td><td>[Display only if answer to C5.1a is “Yes” and answer to B3 is “Yes” and answer to B4c is <u>not</u> “0”.]<br>If you were to treat a patient with mUC with ECOG PS 0-1 and no other comorbidities affecting treatment decision, for which of the following audiometric hearing loss grades would you prescribe <u>split-dose cisplatin</u> rather than standard-dose cisplatin? Please select all that apply.<br>• Grade 1: subjective change in hearing in absence of documented hearing loss<br>• Grade 2: hearing loss but hearing aid or intervention not indicated<br>• Grade 3: hearing loss with hearing aid or intervention indicated<br>• Grade 4: bilateral decrease in hearing to >80 dB HL at 2 kHz and above; nonserviceable hearing.<br><br>☐ Hearing loss grade 1<br>☐ Hearing loss grade 2<br>☐ Hearing loss grade 3<br>☐ Hearing loss grade 4<br>☐ I would not prescribe split-dose cisplatin in any of these scenarios [exclusive]</td></tr>
<tr><td>C1a</td><td>Do you consider patient’s age when deciding on the type of platinum-based chemotherapy in a patient with mUC with ECOG PS 0-1 and no other comorbidities affecting treatment decision?<br>O (1) Yes<br>O (0) No</td></tr>
<tr><td>C1b</td><td>[Display only if the answer to C1a is “Yes” and answer to B3 is “Yes” and answer to B4c is <u>not</u> “0”.]<br>If you were to treat a patient with mUC with ECOG PS 0-1 without comorbidities affecting treatment decision, in which age range would you prescribe <u>split-dose cisplatin</u> rather than standard-dose cisplatin?<br><br>
<table>
<tr><th>Lower threshold:</th><th></th><th>Upper threshold:</th></tr>
<tr><td>Dropdown:<br>O (60) ≥60 years<br>O (65) ≥65 years<br>O (70) ≥70 years<br>O (75) ≥75 years<br>O (80) ≥80 years<br>O (85) ≥85 years<br>O (90) ≥90 years<br>O (99) Other: ≥___________ years<br>O (0) No lower age threshold</td><td>–</td><td>Dropdown:<br>O (65) ≤65 years<br>O (70) ≤70 years<br>O (75) ≤75 years<br>O (80) ≤80 years<br>O (85) ≤85 years<br>O (90) ≤90 years<br>O (95) ≤95 years<br>O (99) Other: ≤___________ years<br>O (0) No upper age threshold</td></tr>
</table>
[Upper threshold must be higher than lower threshold.]</td></tr>
<tr><td>C6</td><td>[Display only if the answer to B3 is “Yes”.]<br>Apart from the patient’s characteristics, what other factors do you consider in your decision to prescribe a split-dose cisplatin regimen to mUC patients? Please select all that apply.<br>☐ (1) Patient preference<br>☐ (2) Treatment reimbursement<br>☐ (3) Consistency of supportive care medications across treatment days<br>☐ (4) Limited availability of/access to alternative treatments<br>☐ (5) Access to subsequent maintenance immune checkpoint inhibitors after first-line platinum-based chemotherapy<br>☐ (6) Chair time/availability of resources<br>☐ (0) None [exclusive]<br>☐ (99) Other, please specify: _______________</td></tr>
<tr><td></td><td>[Print text below]<br>The following questions also refer to the management of patients with <u>unresectable locally advanced or metastatic urothelial cancer (referred to as mUC</u>).</td></tr>
</table>

| No. | Item |
|---|---|
| C7 | *Please note: The following question will be asked 3 times, for patients with differing creatinine clearance levels (first 45-50 mL/min, then 50-55 mL/min, last 55-60 mL/min).*<br><br>Consider a patient with mUC, a creatinine clearance of 45-50 mL/min, ECOG PS 0-1, and no other comorbidities affecting treatment decision. Which regimen(s) would be your first, second, and third choice for mUC first-line treatment? **Only consider treatments available in your clinical practice.** Please rank the following from 1-3, if applicable:<br>(1) *[Drop-down menu with 1-3 & "-"]* Standard-dose cisplatin regimen<br>(2) *[Drop-down menu with 1-3 & "-"]* Split-dose cisplatin regimen<br>(3) *[Drop-down menu with 1-3 & "-"]* Carboplatin regimen<br>(4) *[Drop-down menu with 1-3 & "-"]* Other chemotherapy regimen<br>(5) *[Drop-down menu with 1-3 & "-"]* Single-agent immune checkpoint inhibitor<br>(6) *[Drop-down menu with 1-3 & "-"]* Pembrolizumab and enfortumab vedotin-ejfv<br>(7) *[Drop-down menu with 1-3 & "-"]* Other, please specify: ____________<br>*[Each rank number can only be selected once. Default selection is "-". It is possible to only select rank 1 or rank 1 and 2.]* |
| C7a | Consider a patient with mUC, a creatinine clearance of 50-55 mL/min, ECOG PS 0-1, and no other comorbidities affecting treatment decision. Which regimen(s) would be your first, second, and third choice for mUC first-line treatment? **Only consider treatments available in your clinical practice.** Please rank the following from 1-3, if applicable:<br>(1) *[Drop-down menu with 1-3 & "-"]* Standard-dose cisplatin regimen<br>(2) *[Drop-down menu with 1-3 & "-"]* Split-dose cisplatin regimen<br>(3) *[Drop-down menu with 1-3 & "-"]* Carboplatin regimen<br>(4) *[Drop-down menu with 1-3 & "-"]* Other chemotherapy regimen<br>(5) *[Drop-down menu with 1-3 & "-"]* Single-agent immune checkpoint inhibitor<br>(6) *[Drop-down menu with 1-3 & "-"]* Pembrolizumab and enfortumab vedotin-ejfv<br>(7) *[Drop-down menu with 1-3 & "-"]* Other, please specify: ____________<br>*[Each rank number can only be selected once. Default selection is "-".It is possible to only select rank 1 or rank 1 and 2.]* |
| C7b | Consider a patient with mUC, a creatinine clearance of 55-60 mL/min, ECOG PS 0-1, and no other comorbidities affecting treatment decision. Which regimen(s) would be your first, second, and third choice for mUC first-line treatment? **Only consider treatments available in your clinical practice.** Please rank the following from 1-3, if applicable:<br>(1) *[Drop-down menu with 1-3 & "-"]* Standard-dose cisplatin regimen<br>(2) *[Drop-down menu with 1-3 & "-"]* Split-dose cisplatin regimen<br>(3) *[Drop-down menu with 1-3 & "-"]* Carboplatin regimen<br>(4) *[Drop-down menu with 1-3 & "-"]* Other chemotherapy regimen<br>(5) *[Drop-down menu with 1-3 & "-"]* Single-agent immune checkpoint inhibitor<br>(6) *[Drop-down menu with 1-3 & "-"]* Pembrolizumab and enfortumab vedotin-ejfv<br>(7) *[Drop-down menu with 1-3 & "-"]* Other, please specify: ____________<br>*[Each rank number can only be selected once. Default selection is "-".It is possible to only select rank 1 or rank 1 and 2.]* |
| | *[Print text below]*<br>The following questions refer to the management of patients with unresectable locally advanced or metastatic urothelial cancer (referred to as mUC) with a creatinine clearance of 45-55 mL/min. |
| C8a | In your experience, how does the effectiveness of split-dose cisplatin compare to that of standard-dose cisplatin in patients with mUC and a creatinine clearance of 45-55 mL/min, ECOG PS 0-1, and no other comorbidities affecting treatment decision?<br>O (1) Much better<br>O (2) A little better<br>O (3) No difference<br>O (4) A little worse<br>O (5) Much worse<br>O (97) Not enough experience with split-dose cisplatin to answer this question |

| No. | Item |
|---|---|
| C8 | In your experience, how does the tolerability of split-dose cisplatin compare to that of standard-dose cisplatin in patients with mUC and a creatinine clearance of 45-55 mL/min, ECOG PS 0-1, and no other comorbidities affecting treatment decision?<br>O (1) Much better<br>O (2) A little better<br>O (3) No difference<br>O (4) A little worse<br>O (5) Much worse<br>O (97) Not enough experience with split-dose cisplatin to answer this question |
| C9 | In your experience, how does the effectiveness of split-dose cisplatin compare to that of carboplatin in patients with mUC and a creatinine clearance of 45-55 mL/min, ECOG PS 0-1, and no other comorbidities affecting treatment decision?<br>O (1) Much better<br>O (2) A little better<br>O (3) No difference<br>O (4) A little worse<br>O (5) Much worse<br>O (97) Not enough experience with split-dose cisplatin to answer this question |
| C10 | In your experience, how does the tolerability of split-dose cisplatin compare to that of carboplatin in patients with mUC and a creatinine clearance of 45-55 mL/min, ECOG PS 0-1, and no other comorbidities affecting treatment decision?<br>O (1) Much better<br>O (2) A little better<br>O (3) No difference<br>O (4) A little worse<br>O (5) Much worse<br>O (97) Not enough experience with split-dose cisplatin to answer this question |
| C11 | How strongly do you agree with the following statement:<br>"Split-dose cisplatin regimens are a reasonable treatment option for patients with mUC and a creatinine clearance of 45-55 mL/min, ECOG PS 0-1, and no other comorbidities affecting treatment decision."<br>O (1) Strongly agree<br>O (2) Agree<br>O (3) Neither agree nor disagree<br>O (4) Disagree<br>O (5) Strongly disagree<br>O (97) Unsure |
| C12 | Please enter any additional comments:<br>____________________<br>*[Answer to C12 is optional.]* |
| *[Show text below]*<br>Thank you for participating in this survey. We appreciate your time, and your responses are very valuable to us. | |

**Supplemental Table S2. Participant selection and exclusion reasons.**

| | Total | USA | India | Brazil | Germany | France | UK | Italy | Spain | Canada | Australia |
|---|---|---|---|---|---|---|---|---|---|---|---|
| **Total number of physicians who initiated the survey** | **1254** | **275** | **169** | **159** | **87** | **92** | **86** | **106** | **193** | **47** | **40** |
| Terminate S1b (Not board eligible in the US) | 2 (0.2%) | 2 (0.7%) | N/A | N/A | N/A | N/A | N/A | N/A | N/A | N/A | N/A |
| Terminate S2 (Specialty is not oncologist/urologist) | 77 (6.1%) | 33 (12.0%) | 8 (4.7%) | 8 (5.0%) | 0 | 6 (6.5%) | 4 (4.7%) | 4 (3.8%) | 3 (1.6%) | 7 (14.9%) | 4 (10.0%) |
| Terminate S3 (<2 years since the end of medical specialist training) | 19 (1.5%) | 3 (1.1%) | 1 (0.6%) | 5 (3.1%) | 1 (1.1%) | 1 (1.1%) | 1 (1.2%) | 2 (1.9%) | 2 (1.0%) | 2 (4.3%) | 1 (2.5%) |
| Terminate S4 (treated <10 patients with UC during the past 12 months) | 31 (2.5%) | 6 (2.2%) | 2 (1.2%) | 14 (8.8%) | 2 (2.3%) | 0 | 0 | 0 | 2 (1.0%) | 3 (6.4%) | 2 (5.0%) |
| Terminate S5 (treated <5 patients with la/mUC during the past 12 months) | 25 (2.0%) | 7 (2.5%) | 4 (2.4%) | 11 (6.9%) | 0 | 1 (1.1%) | 0 | 0 | 2 (1.0%) | 0 | 0 |
| Terminate S6 (little/no responsibility for prescribing and overseeing systemic anticancer therapy) | 15 (1.2%) | 2 (0.7%) | 0 | 1 (0.6%) | 0 | 0 | 0 | 1 (0.9%) | 10 (5.2%) | 0 | 1 (2.5%) |
| Terminated: over quota (specialty)[a] | 28 (2.2%) | 13 (4.7%) | 0 | 3 (1.9%) | 5 (5.7%) | 1 (1.1%) | 3 (3.5%) | 0 | 1 (0.5%) | 0 | 2 (5.0%) |
| Terminated: over quota (practice location)[a] | 159 (12.7%) | 31 (11.3%) | 0 | 4 (2.5%) | 0 | 7 (7.6%) | 0 | 23 (21.7%) | 94 (48.7%) | 0 | 0 |
| Incomplete screener | 52 (4.1%) | 25 (9.1%) | 6 (3.6%) | 8 (5.0%) | 3 (3.4%) | 1 (1.1%) | 2 (2.3%) | 0 | 3 (1.6%) | 0 | 4 (10.0%) |
| Incomplete survey | 55 (4.4%) | 2 (0.7%) | 41 (24.3%) | 5 (3.1%) | 1 (1.1%) | 0 | 1 (1.2%) | 1 (0.9%) | 1 (0.5%) | 2 (4.3%) | 1 (2.5%) |
| **Completed the survey** | **791 (63.1%)** | **151 (54.9%)** | **107 (63.3%)** | **100 (62.9%)** | **75 (86.2%)** | **75 (81.5%)** | **75 (87.2%)** | **75 (70.8%)** | **75 (38.9%)** | **33 (70.2%)** | **25 (62.5%)** |

HCP, healthcare provider; la/mUC, locally advanced or metastatic urothelial cancer; N/A, not applicable; UC, urothelial cancer.
Table shows n of physicians who initiated the survey, those excluded at each exclusion step, and the final number of patients who completed the survey.

[a] Quotas for practice setting and specialty were set per country to ensure data collection in a sample of HCPs representative of the setting in which patients with UC are treated with systemic therapies.

**Supplemental Table S3. Physician demographics and characteristics by country.**

| | Total | USA | India | Brazil | Germany | France | UK | Italy | Spain | Canada | Australia |
|---|---|---|---|---|---|---|---|---|---|---|---|
| | **N=791** | **n=151** | **n=107** | **n=100** | **n=75** | **n=75** | **n=75** | **n=75** | **n=75** | **n=33** | **n=25** |
| **Age, years** | | | | | | | | | | | |
| Mean (SD) | 43.2 (9.3) | 44.4 (10.6) | 39.7 (7.4) | 39.2 (6.7) | 48.0 (9.2) | 44.3 (8.5) | 46.5 (8.2) | 40.6 (9.6) | 42.7 (8.3) | 47.7 (10.8) | 43.5 (9.4) |
| Median [IQR] | 41 [36-50] | 44 [37-51] | 39 [35-42] | 38 [35-42] | 48 [41-56] | 45 [38-50] | 46 [40-54] | 37 [34-48] | 41 [37-49] | 48 [40-56] | 40 [35-50] |
| **Years in clinical practice[a]** | | | | | | | | | | | |
| Mean (SD) | 13.2 (7.3) | 14.2 (7.7) | 10.8 (6.9) | 9.8 (5.7) | 16.6 (7.2) | 14.8 (6.6) | 13.3 (5.9) | 12.0 (7.5) | 14.0 (6.7) | 17.1 (7.9) | 10.9 (7.9) |
| Median [IQR] | 12 [7-18] | 12 [8-20] | 9 [7-14] | 8 [6-12] | 15 [12-22] | 15 [9-20] | 14 [9-16] | 10 [6-17] | 14 [8-20] | 16 [11-25] | 8 [4-16] |
| **Gender, n (%)** | | | | | | | | | | | |
| Female | 210 (26.5) | 21 (13.9) | 16 (15.0) | 46 (46.0) | 15 (20.0) | 19 (25.3) | 14 (18.7) | 33 (44.0) | 29 (38.7) | 11 (33.3) | 6 (24.0) |
| Male | 578 (73.1) | 129 (85.4) | 91 (85.0) | 54 (54.0) | 60 (80.0) | 56 (74.7) | 60 (80.0) | 42 (56.0) | 46 (61.3) | 22 (66.7) | 18 (72.0) |
| Other | 3 (0.4) | 1 (0.7) | 0 | 0 | 0 | 0 | 1 (1.3) | 0 | 0 | 0 | 1 (4.0) |
| **Medical specialty, n (%)** | | | | | | | | | | | |
| Medical oncologist | 725 (91.7) | 151 (100) | 91 (85.0) | 100 (100) | 35 (46.7) | 75 (100) | 75 (100) | 75 (100) | 65 (86.7) | 33 (100) | 25 (100) |
| Urologist | 66 (8.3) | 0 | 16 (15.0) | 0 | 40 (53.3) | 0 | 0 | 0 | 10 (13.3) | 0 | 0 |
| **Practice setting, n (%)** | | | | | | | | | | | |
| Private | 271 (34.3) | 61 (40.4) | 90 (84.1) | 88 (88.0) | 15 (20.0) | 7 (9.3) | 1 (1.3) | 3 (4.0) | 4 (5.3) | 0 | 2 (8.0) |
| Public | 436 (55.1) | 62 (41.1) | 13 (12.1) | 4 (4.0) | 42 (56.0) | 65 (86.7) | 67 (89.3) | 72 (96.0) | 68 (90.7) | 30 (90.9) | 13 (52.0) |
| I spend equal time in either setting | 84 (10.6) | 28 (18.5) | 4 (3.7) | 8 (8.0) | 18 (24.0) | 3 (4.0) | 7 (9.3) | 0 | 3 (4.0) | 3 (9.1) | 10 (40.0) |
| **Principal practice location, n (%)** | | | | | | | | | | | |
| Academic/teaching hospital or specialized cancer center | 465 (58.8) | 78 (51.7) | 42 (39.3) | 44 (44.0) | 33 (44.0) | 41 (54.7) | 67 (89.3) | 47 (62.7) | 67 (89.3) | 25 (75.8) | 21 (84.0) |
| Community/nonteaching hospital | 212 (26.8) | 43 (28.5) | 64 (59.8) | 6 (6.0) | 12 (16.0) | 33 (44.0) | 8 (10.7) | 28 (37.3) | 8 (10.7) | 8 (24.2) | 2 (8.0) |
| Office-based practice | 114 (14.4) | 30 (19.9) | 1 (0.9) | 50 (50.0) | 30 (40.0) | 1 (1.3) | 0 | 0 | 0 | 0 | 2 (8.0) |
| **Hospital/center size, n (%)** | | | | | | | | | | | |
| Large (≥500 beds) | 404 (51.1) | 75 (49.7) | 40 (37.4) | 22 (22.0) | 34 (45.3) | 53 (70.7) | 57 (76.0) | 43 (57.3) | 48 (64.0) | 17 (51.5) | 15 (60.0) |
| Medium (100-499 beds) | 231 (29.2) | 43 (28.5) | 46 (43.0) | 26 (26.0) | 8 (10.7) | 20 (26.7) | 17 (22.7) | 26 (34.7) | 26 (34.7) | 13 (39.4) | 6 (24.0) |

| | | | | | | | | | | | |
|---|---|---|---|---|---|---|---|---|---|---|---|
| Small (<100 beds) | 36 (4.6) | 3 (2.0) | 19 (17.8) | 2 (2.0) | 1 (1.3) | 1 (1.3) | 1 (1.3) | 3 (4.0) | 1 (1.3) | 3 (9.1) | 2 (8.0) |
| Don't know/unsure | 6 (0.8) | 0 | 1 (0.9) | 0 | 2 (2.7) | 0 | 0 | 3 (4.0) | 0 | 0 | 0 |
| NA (office-based practice) | 114 (14.4) | 30 (19.9) | 1 (0.9) | 50 (50.0) | 30 (40.0) | 1 (1.3) | 0 | 0 | 0 | 0 | 2 (8.0) |
| **Number of monthly new (first-time) patients with UC** | | | | | | | | | | | |
| Mean (SD) | 24.9 (44.8) | 31.1 (49.3) | 11.7 (25.8) | 11.7 (15.1) | 29.5 (49.6) | 23.4 (36.6) | 40.3 (80.6) | 32.0 (45.9) | 34.6 (39.1) | 12.9 (14.7) | 8.0 (12.6) |
| Median [IQR] | 10 [5-28] | 14 [5-30] | 5 [3-10] | 6 [4-15] | 15 [6-33] | 15 [8-28] | 16 [10-40] | 18 [10-35] | 20 [11-45] | 8 [5-13] | 4 [2-8] |
| **Number of monthly new (first-time) and follow-up patients with UC** | | | | | | | | | | | |
| Mean (SD) | 42.0 (59.2) | 51.8 (71.2) | 21.4 (34.9) | 23.3 (23.8) | 43.2 (59.5) | 42.2 (60.3) | 58.8 (90.5) | 56.5 (58.6) | 57.5 (56.0) | 25.9 (21.8) | 23.7 (30.6) |
| Median [IQR] | 25 [12-50] | 25 [15-58] | 10 [6-23] | 15 [10-26] | 25 [14-50] | 30 [19-50] | 35 [20-52] | 40 [22-68] | 40 [24-70] | 18 [10-35] | 10 [8-20] |
| **Number of monthly new (first time) patients with la/mUC treated** | | | | | | | | | | | |
| Mean (SD) | 28.5 (44.5) | 36.6 (57.1) | 10.7 (16.6) | 16.2 (18.1) | 20.1 (21.9) | 34.2 (59.4) | 40.6 (65.8) | 43.3 (44.6) | 38.3 (40.3) | 18.8 (19.4) | 14.8 (17.3) |
| Median [IQR] | 15 [8-30] | 20 [10-37] | 5 [3-10] | 10 [5-20] | 15 [5-21] | 20 [10-45] | 25 [14-40] | 30 [18-50] | 25 [14-50] | 12 [8-20] | 8 [4-15] |
| **Enrollment of patients with UC in clinical trials, n (%)** | | | | | | | | | | | |
| Yes | 395 (49.9) | 73 (48.3) | 26 (24.3) | 47 (47.0) | 29 (38.7) | 34 (45.3) | 41 (54.7) | 51 (68.0) | 56 (74.7) | 19 (57.6) | 19 (76.0) |
| No | 396 (50.1) | 78 (51.7) | 81 (75.7) | 53 (53.0) | 46 (61.3) | 41 (54.7) | 34 (45.3) | 24 (32.0) | 19 (25.3) | 14 (42.4) | 6 (24.0) |
| **Guidelines considered for the treatment of UC, n (%)[b]** | | | | | | | | | | | |
| EAU | 271 (34.3) | 21 (13.9) | 33 (30.8) | 22 (22.0) | 47 (62.7) | 43 (57.3) | 21 (28.0) | 36 (48.0) | 37 (49.3) | 5 (15.2) | 6 (24.0) |
| ESMO | 476 (60.2) | 32 (21.2) | 39 (36.4) | 72 (72.0) | 41 (54.7) | 67 (89.3) | 60 (80.0) | 66 (88.0) | 68 (90.7) | 13 (39.4) | 18 (72.0) |
| NCCN | 490 (61.9) | 132 (87.4) | 85 (79.4) | 80 (80.0) | 12 (16.0) | 28 (37.3) | 25 (33.3) | 39 (52.0) | 48 (64.0) | 22 (66.7) | 19 (76.0) |
| AUA/SUO | 115 (14.5) | 31 (20.5) | 11 (10.3) | 11 (11.0) | 7 (9.3) | 9 (12.0) | 11 (14.7) | 14 (18.7) | 11 (14.7) | 7 (21.2) | 3 (12.0) |
| ASCO | 381 (48.2) | 88 (58.3) | 20 (18.7) | 81 (81.0) | 18 (24.0) | 34 (45.3) | 18 (24.0) | 36 (48.0) | 50 (66.7) | 22 (66.7) | 14 (56.0) |
| National guideline(s) | 143 (18.1) | 0 | 4 (3.7) | 26 (26.0) | 17 (22.7) | 14 (18.7) | 21 (28.0) | 31 (41.3) | 20 (26.7) | 6 (18.2) | 4 (16.0) |
| Other international guideline(s) | 6 (0.8) | 0 | 1 (0.9) | 1 (1.0) | 0 | 0 | 2 (2.7) | 1 (1.3) | 1 (1.3) | 0 | 0 |
| Institutional guideline(s) | 100 (12.6) | 9 (6.0) | 11 (10.3) | 14 (14.0) | 8 (10.7) | 5 (6.7) | 25 (33.3) | 7 (9.3) | 8 (10.7) | 7 (21.2) | 6 (24.0) |

| | | | | | | | | | | | |
|---|---|---|---|---|---|---|---|---|---|---|---|
| None[c] | 11 (1.4) | 2 (1.3) | 0 | 1 (1.0) | 3 (4.0) | 0 | 4 (5.3) | 0 | 0 | 0 | 1 (4.0) |
| **Method to assess kidney function in patients with UC being considered for platinum-based chemotherapy, n (%)[b]** | | | | | | | | | | | |
| Calculated creatinine clearance | 680 (86.0) | 118 (78.1) | 81 (75.7) | 90 (90.0) | 64 (85.3) | 71 (94.7) | 67 (89.3) | 66 (88.0) | 69 (92.0) | 31 (93.9) | 23 (92.0) |
| Measured creatinine clearance | 196 (24.8) | 49 (32.5) | 10 (9.3) | 20 (20.0) | 33 (44.0) | 13 (17.3) | 21 (28.0) | 23 (30.7) | 16 (21.3) | 9 (27.3) | 2 (8.0) |
| Measured GFR | 251 (31.7) | 66 (43.7) | 12 (11.2) | 17 (17.0) | 23 (30.7) | 24 (32.0) | 39 (52.0) | 22 (29.3) | 33 (44.0) | 9 (27.3) | 6 (24.0) |
| Serum creatinine value | 338 (42.7) | 81 (53.6) | 42 (39.3) | 40 (40.0) | 42 (56.0) | 24 (32.0) | 23 (30.7) | 32 (42.7) | 34 (45.3) | 15 (45.5) | 5 (20.0) |
| Other | 5 (0.6) | 0 | 2 (1.9) | 0 | 1 (1.3) | 1 (1.3) | 0 | 0 | 0 | 1 (3.0) | 0 |
| **Formula used to calculate creatinine clearance, n (%)[d]** | **n=680** | **n=118** | **n=81** | **n=90** | **n=64** | **n=71** | **n=67** | **n=66** | **n=69** | **n=31** | **n=23** |
| Cockcroft-Gault equation | 462 (67.9) | 81 (68.6) | 72 (88.9) | 62 (68.9) | 30 (46.9) | 26 (36.6) | 57 (85.1) | 51 (77.3) | 38 (55.1) | 27 (87.1) | 18 (78.3) |
| Modification of Diet in Renal Disease equation | 82 (12.1) | 8 (6.8) | 5 (6.2) | 11 (12.2) | 12 (18.8) | 27 (38.0) | 5 (7.5) | 5 (7.6) | 7 (10.1) | 1 (3.2) | 1 (4.3) |
| Chronic Kidney Disease Epidemiology Collaboration equation | 99 (14.6) | 23 (19.5) | 2 (2.5) | 16 (17.8) | 8 (12.5) | 16 (22.5) | 1 (1.5) | 7 (10.6) | 20 (29.0) | 2 (6.5) | 4 (17.4) |
| Jelliffe equation | 4 (0.6) | 0 | 1 (1.2) | 0 | 0 | 1 (1.4) | 1 (1.5) | 1 (1.5) | 0 | 0 | 0 |
| Wright equation | 6 (0.9) | 2 (1.7) | 0 | 0 | 0 | 0 | 3 (4.5) | 1 (1.5) | 0 | 0 | 0 |
| Unsure | 24 (3.5) | 2 (1.7) | 1 (1.2) | 1 (1.1) | 13 (20.3) | 1 (1.4) | 0 | 1 (1.5) | 4 (5.8) | 1 (3.2) | 0 |
| Other | 3 (0.4) | 2 (1.7) | 0 | 0 | 1 (1.6) | 0 | 0 | 0 | 0 | 0 | 0 |
| **Prescribes split-dose cisplatin (any setting), n (%)** | 670 (84.7) | 136 (90.1) | 82 (76.6) | 67 (67.0) | 68 (90.7) | 70 (93.3) | 61 (81.3) | 70 (93.3) | 65 (86.7) | 32 (97.0) | 19 (76.0) |
| | **n=670 [e]** | **n=136** | **n=82** | **n=67** | **n=68** | **n=70** | **n=61** | **n=70** | **n=65** | **n=32** | **n=19** |
| **Percentage of patients treated with split-dose cisplatin in the neoadjuvant setting** | | | | | | | | | | | |
| Mean (SD) | 40.9 (26.3) | 40.7 (22.8) | 49.0 (30.1) | 70.0 (25.5) | 26.4 (21.8) | 35.2 (20.9) | 39.5 (24.6) | 35.9 (20.4) | 37.0 (23.4) | 31.7 (19.1) | 27.8 (28.3) |
| Median [IQR] | 34 [20-56] | 40 [25-50] | 50 [20-75] | 70 [50-98] | 20 [10-30] | 30 [20-50] | 31 [20-50] | 30 [20-45] | 30 [20-50] | 30 [20-50] | 20 [10-32] |

| | | | | | | | | | | | |
|---|---|---|---|---|---|---|---|---|---|---|---|
| **Percentage of patients treated with split-dose cisplatin in the adjuvant setting** | | | | | | | | | | | |
| Mean (SD) | 39.5 (26.0) | 39.7 (23.0) | 47.3 (31.1) | 64.4 (27.8) | 29.8 (24.9) | 35.2 (21.4) | 40.4 (23.9) | 32.3 (18.8) | 35.0 (22.4) | 29.9 (19.9) | 21.7 (21.5) |
| Median [IQR] | 35 [20-54] | 38 [25-50] | 50 [20-75] | 65 [50-83] | 25 [10-31] | 30 [20-50] | 35 [20-55] | 30 [20-40] | 33 [20-50] | 28 [19-41] | 20 [8-20] |
| **Percentage of patients treated with split-dose cisplatin in the advanced setting** | | | | | | | | | | | |
| Mean (SD) | 43.0 (26.8) | 41.7 (23.9) | 54.4 (30.7) | 70.2 (27.9) | 30.9 (23.7) | 39.2 (23.3) | 40.5 (23.7) | 38.3 (21.9) | 35.2 (20.9) | 33.4 (22.5) | 33.3 (22.6) |
| Median [IQR] | 40 [20-60] | 35 [25-57] | 50 [30-80] | 80 [50,100] | 25 [15-40] | 30 [20-50] | 40 [20-50] | 35 [25-50] | 30 [20-50] | 30 [19-46] | 33 [15-48] |

ASCO, American Society of Clinical Oncology; AUA, American Urological Association; EAU, European Association of Urology; ESMO, European Society for Medical Oncology; GC, gemcitabine + cisplatin; GFR, glomerular filtration rate; IQR, interquartile range; la/mUC, locally advanced or metastatic urothelial cancer; N/A, not applicable; NCCN, National Comprehensive Cancer Network; SD, standard deviation; SUO, Society of Urologic Oncology; UC, urothelial cancer.

[a] Years in clinical practice since the end of medical specialist training. **[b]** Multiple answers possible. [c] "I do not consider specific guidelines for the treatment of [patients with UC]." [d] Assessed only in respondents who calculated creatinine clearance. **[e]** Questions asked to physicians who indicated they generally prescribe split-dose cisplatin to patients with UC.

**Supplemental Table S4. Details on split-dose cisplatin use in la/mUC by geographic region.**

| | **Total**<br><br>**N=660** | **Europe[a]**<br><br>**N=330** | **North America[b]**<br>**N=164** | **Brazil**<br><br>**N=67** | **India**<br><br>**N=81** | **Australia**<br><br>**N=18** | ***P value*** |
|---|---|---|---|---|---|---|---|
| **Within the unresectable locally advanced or metastatic setting, in which line(s) of treatment do you consider using split-dose cisplatin? (multiple answers possible)** | | | | | | | |
| 1L | 548 (83.0) | 125 (76.2) | 282 (85.5) | 64 (95.5) | 62 (76.5) | 15 (83.3) | 0.005 |
| 2L | 340 (51.5) | 105 (64.0) | 173 (52.4) | 31 (46.3) | 26 (32.1) | 5 (27.8) | <0.001 |
| 3L | 168 (25.5) | 55 (33.5) | 85 (25.8) | 14 (20.9) | 10 (12.3) | 4 (22.2) | 0.008 |
| **Which split-dose cisplatin regimens do you use in patients with la/mUC? (top five regimens; multiple answers possible)** | | | | | | | |
| GC 35 mg/m$^2$, days 1 + 8 of 21-day cycles | 378 (57.3) | 185 (56.1) | 92 (56.1) | 48 (71.6) | 39 (48.1) | 14 (77.8) | 0.542 |
| GC 25 mg/m$^2$, days 1 + 8 of 21-day cycles | 86 (13.0) | 46 (13.9) | 17 (10.4) | 9 (13.4) | 14 (17.3) | 0 (0.0) | |
| GC 35 mg/m$^2$, days 1 + 8 of 28-day cycles | 36 (5.5) | 19 (5.8) | 10 (6.1) | 0 (0.0) | 6 (7.4) | 1 (5.6) | |
| GC 35 mg/m$^2$, days 1 + 2 of 21-day cycles | 25 (3.8) | 9 (2.7) | 5 (3.0) | 3 (4.5) | 6 (7.4) | 2 (11.1) | |
| GC 35 mg/m$^2$, days 1 + 15 of 21-day cycles | 20 (3.0) | 12 (3.6) | 6 (3.7) | 0 (0.0) | 2 (2.5) | 0 (0.0) | |
| **In patients with la/mUC, do you routinely use avelumab as first-line maintenance if there is no disease progression during or after split-dose cisplatin-based chemotherapy?** | | | | | | | |
| Yes | 531 (80.5) | 133 (81.1) | 295 (89.4) | 62 (92.5) | 23 (28.4) | 18 (100) | <0.001 |
| No | 129 (19.5) | 31 (18.9) | 35 (10.6) | 5 (7.5) | 58 (71.6) | 0 (0) | |

1L, first line; 2L, second line; 3L, third line; GC, gemcitabine + cisplatin; la/mUC, locally advanced or metastatic urothelial cancer.

[a] Europe includes respondents from France, Germany, Italy, Spain, and the UK. [b] North America includes respondents from USA and Canada.

## Supplemental Table S5. Reasons for not using split-dose cisplatin.

| | **N=121** |
|---|---|
| **What is your rationale for not using split-dose cisplatin? (multiple answers possible)** | |
| Lack of evidence for efficacy | 37 (30.6) |
| Clinical experience of poor efficacy | 23 (19.0) |
| Concerns regarding toxicity in patients with poor renal function | 34 (28.1) |
| Not part of institutional treatment protocol/guidance | 55 (45.5) |
| Not familiar with this regimen | 32 (26.4) |
| Other | 10 (8.3) |

**Supplemental Table S6. Predictors of prescribing split-dose cisplatin to patients with UC across countries: univariable analysis.**

| Covariate | OR (95% CI) | *P* value |
|---|---|---|
| Specialty: oncologist | 1 (ref) | |
| Specialty: urologist | 0.58 (0.32-1.11) | 0.083 |
| Practice years (per decade) [a] | 1.67 (1.24-2.27) | 0.001 |
| Age (per decade)[a] | 1.40 (1.12-1.75) | 0.003 |
| Practice setting: private | 1 (ref) | |
| Practice setting: public | 2.34 (1.56-3.53) | 0.001 |
| Practice setting: I spend equal time in either setting | 2.88 (1.39-6.76) | 0.008 |
| Location: academic/teaching hospital or specialized cancer center | 1 (ref) | |
| Location: community/non-teaching/district hospital | 0.82 (0.53-1.30) | 0.390 |
| Location: office-based practice | 0.72 (0.42-1.26) | 0.233 |
| Gender: female | 1 (ref) | |
| Gender: male | 0.99 (0.63-1.52) | 0.956 |
| Total number of patients with UC per month (per 10 patients) | 1.09 (1.03-1.17) | 0.007 |
| Region: Europe | 1 (ref) | |
| Region: North America | 1.29 (0.72-2.43) | 0.412 |
| Region: Brazil | 0.25 (0.15-0.42) | 0.001 |
| Region: India | 0.40 (0.23-0.71) | 0.001 |
| Region: Australia | 0.39 (0.15-1.12) | 0.057 |

OR, odds ratio; UC, urothelial cancer.

[a] Only variables without obvious correlation were included in the multivariable model to avoid multicollinearity. A strong positive correlation was observed between age and years of practice (rs=0.80, p<0.001). Therefore, age was not considered for the multivariable model.

**Supplemental Table S7. Predictors of prescribing split-dose cisplatin to patients with UC by geographic region: multivariable logistic regression model.**

| Covariate | OR (95% CI) | *P* value |
|---|---|---|
| **Europe[a] (N=375)** | | |
| Specialty: oncologist | 1 (ref) | |
| Specialty: urologist | 0.47 (0.18-1.39) | 0.142 |
| Practice years (per decade) | 1.78 (1.06-3.10) | 0.034 |
| Practice setting: private | 1 (ref) | |
| Practice setting: public | 0.69 (0.15-2.17) | 0.569 |
| Practice setting: I spend equal time in either setting | NA (unstable estimate)[b] | 0.989 |
| Total number of patients with UC per month (per 10 patients) | 1.02 (0.97-1.10) | 0.503 |
| **North America[c] (N=184)** | | |
| Specialty: oncologist[d] | - | - |
| Specialty: urologist[d] | - | - |
| Practice years (per decade) | 0.75 (0.39-1.46) | 0.388 |
| Practice setting: private | 1 (ref) | |
| Practice setting: public | 2.30 (0.71-8.14) | 0.172 |
| Practice setting: I spend equal time in either setting | 0.58 (0.13-3.18) | 0.501 |
| Total number of patients with UC per month (per 10 patients) | 1.43 (1.08-2.25) | 0.055 |
| **Brazil (N=100)** | | |
| Specialty: oncologist[d] | - | - |
| Specialty: urologist[d] | - | - |
| Practice years (per decade) | 1.17 (0.56-2.62) | 0.683 |
| Practice setting: private | 1 (ref) | |
| Practice setting: public | NA (unstable estimate)[b] | 0.989 |
| Practice setting: I spend equal time in either setting | 0.84 (0.19-4.37) | 0.825 |
| Total number of patients with UC per month (per 10 patients) | 1.02 (0.85-1.24) | 0.850 |
| **India (N=107)** | | |
| Specialty: oncologist[d] | 1 (ref) | |
| Specialty: urologist[d] | 0.09 (0.02-0.34) | 0.001 |
| Practice years (per decade) | 2.79 (1.13-8.61) | 0.047 |
| Practice setting: private | 1 (ref) | |
| Practice setting: public | 0.69 (0.17-3.50) | 0.619 |
| Practice setting: I spend equal time in either setting | NA (unstable estimate)[b] | 0.993 |
| Total number of patients with UC per month (per 10 patients) | 1.20 (0.95-1.77) | 0.267 |
| **Australia (N=25)** | | |
| Specialty: oncologist[d] | - | - |
| Specialty: urologist[d] | - | - |
| Practice years (per decade) | 0.67 (0.19-2.54) | 0.527 |
| Practice setting: private | 1 (ref) | |
| Practice setting: public | NA (unstable estimate) [b] | 0.994 |
| Practice setting: I spend equal time in either setting | NA (unstable estimate)[b] | 0.995 |
| Total number of patients with UC per month (per 10 patients) | 0.84 (0.55-1.28) | 0.392 |

NA, not available; OR, odds ratio; UC, urothelial cancer.

[a] Europe includes respondents from France, Germany, Italy, Spain, and the UK. [b] The odds ratio could not be reliably estimated due to sparse data, resulting in an unstable estimate. [c] North America includes respondents from USA and Canada. [d] Covariate was excluded due to lack of variability (all respondents were medical oncologists).

**Supplemental Table S8. Consideration of characteristics in physician's decision to prescribe split-dose cisplatin by geographical region.**

| | Overall | Europe[a] | North America[b] | Brazil | India | Australia | *P value* |
|---|---|---|---|---|---|---|---|
| **If you were to treat a patient with mUC with ECOG = 0-1 and no other comorbidities affecting treatment decision, in which range of creatinine clearance would you prescribe split-dose cisplatin rather than standard-dose cisplatin? (Lower Threshold)[c]** | | | | | | | |
| | **(N=635)** | **(N=317)** | **(N=156)** | **(N=65)** | **(N=79)** | **(N=18)** | p=0.019 |
| ≥25 mL/min | 68 (10.7) | 32 (10.1) | 20 (12.8) | 2 (3.1) | 11 (13.9) | 3 (16.7) | |
| ≥30 mL/min | 113 (17.8) | 63 (19.9) | 30 (19.2) | 7 (10.8) | 8 (10.1) | 5 (27.8) | |
| ≥35 mL/min | 94 (14.8) | 46 (14.5) | 24 (15.4) | 4 (6.2) | 18 (22.8) | 2 (11.1) | |
| ≥40 mL/min | 118 (18.6) | 52 (16.4) | 32 (20.5) | 10 (15.4) | 20 (25.3) | 4 (22.2) | |
| ≥45 mL/min | 60 (9.4) | 28 (8.8) | 14 (9.0) | 8 (12.3) | 9 (11.4) | 1 (5.6) | |
| ≥50 mL/min | 106 (16.7) | 58 (18.3) | 20 (12.8) | 18 (27.7) | 7 (8.9) | 3 (16.7) | |
| ≥55 mL/min | 19 (3.0) | 10 (3.2) | 2 (1.3) | 6 (9.2) | 1 (1.3) | 0 (0) | |
| ≥60 mL/min | 43 (6.8) | 20 (6.3) | 10 (6.4) | 9 (13.8) | 4 (5.1) | 0 (0) | |
| No lower creatinine clearance threshold | 14 (2.2) | 8 (2.5) | 4 (2.6) | 1 (1.5) | 1 (1.3) | 0 (0) | |
| **If you were to treat a patient with mUC with ECOG = 0-1 and no other comorbidities affecting treatment decision, in which range of creatinine clearance would you prescribe split-dose cisplatin rather than standard-dose cisplatin? (Lower Threshold)[c]** | | | | | | | |
| | **(N=635)** | **(N=317)** | **(N=156)** | **(N=65)** | **(N=79)** | **(N=18)** | |
| ≥30 mL/min | 111 (17.5) | 53 (16.7) | 29 (18.6) | 6 (9.2) | 14 (17.7) | 9 (50.0) | p=0.002 |
| ≥35 mL/min | 82 (12.9) | 49 (15.5) | 20 (12.8) | 2 (3.1) | 11 (13.9) | 0 (0) | |
| ≥40 mL/min | 89 (14.0) | 39 (12.3) | 28 (17.9) | 8 (12.3) | 13 (16.5) | 1 (5.6) | |
| ≥45 mL/min | 68 (10.7) | 31 (9.8) | 15 (9.6) | 6 (9.2) | 15 (19.0) | 1 (5.6) | |
| ≥50 mL/min | 85 (13.4) | 46 (14.5) | 19 (12.2) | 9 (13.8) | 6 (7.6) | 5 (27.8) | |
| ≥55 mL/min | 28 (4.4) | 14 (4.4) | 8 (5.1) | 4 (6.2) | 2 (2.5) | 0 (0) | |
| ≥60 mL/min | 64 (10.1) | 27 (8.5) | 12 (7.7) | 17 (26.2) | 8 (10.1) | 0 (0) | |
| N/A[d] | 100 (15.7) | 54 (17.0) | 22 (14.1) | 12 (18.5) | 10 (12.7) | 2 (11.1) | |
| **If you were to treat a patient with mUC with ECOG PS = 0-1 without comorbidities affecting treatment decision, in which age range would you prescribe split-dose cisplatin rather than standard-dose cisplatin? (Upper Threshold)[e]** | | | | | | | |
| | **(N=472)** | **(N=242)** | **(N=109)** | **(N=45)** | **(N=64)** | **(N=12)** | |
| ≤65 years | 22 (4.7) | 6 (2.5) | 6 (5.5) | 1 (2.2) | 9 (14.1) | 0 (0) | p<0.001 |
| ≤70 years | 41 (8.7) | 14 (5.8) | 4 (3.7) | 5 (11.1) | 17 (26.6) | 1 (8.3) | |
| ≤75 years | 83 (17.6) | 48 (19.8) | 17 (15.6) | 4 (8.9) | 14 (21.9) | 0 (0) | |
| ≤80 years | 101 (21.4) | 53 (21.9) | 23 (21.1) | 12 (26.7) | 11 (17.2) | 2 (16.7) | |
| ≤85 years | 94 (19.9) | 59 (24.4) | 19 (17.4) | 7 (15.6) | 4 (6.3) | 5 (41.7) | |
| ≤90 years | 41 (8.7) | 20 (8.3) | 15 (13.8) | 3 (6.7) | 3 (4.7) | 0 (0) | |
| ≤95 years | 23 (4.9) | 10 (4.1) | 7 (6.4) | 4 (8.9) | 1 (1.6) | 1 (8.3) | |
| No upper age threshold | 67 (14.2) | 32 (13.2) | 18 (16.5) | 9 (20.0) | 5 (7.8) | 3 (25.0) | |

CrCl, creatinine clearance; ECOG PS, Eastern Cooperative Oncology Group performance status; la/mUC, locally advanced or metastatic urothelial cancer.

[a] Europe includes respondents from France, Germany, Italy, Spain, and the UK. [b] North America includes respondents from USA and Canada. [c] Only asked in respondents who prescribe split-dose cisplatin to mUC patients and consider kidney function in their platinum treatment decision. [d] N/A indicates "I would not prescribe any cisplatin regimen in a patient with ECOG PS 2." [e] Only asked in respondents who prescribe split-dose cisplatin to patients with mUC and consider age in their platinum treatment decision.

**Supplemental Table S9. Preferred regimens for otherwise fit patients with la/mUC with borderline kidney function based on creatinine clearance, by geographical region.**

| | Overall<br>N=791 | Europe[a]<br>N=375 | North America[b]<br>N=184 | Brazil<br>N=100 | India<br>N=107 | Australia<br>N=25 | *P value* |
|---|---|---|---|---|---|---|---|
| **Consider a patient with mUC, a creatinine clearance of 55-60 ml/min, ECOG PS = 0-1, and no other comorbidities affecting treatment decision. Which regimen(s) would be your first, second, and third choice for mUC first-line treatment?** | | | | | | | |
| Standard-dose cisplatin | | | | | | | p=0.039 |
| 1 | 347 (43.9%) | 175 (46.7%) | 63 (34.2%) | 38 (38.0%) | 56 (52.3%) | 15 (60.0%) | |
| 2 | 147 (18.6%) | 61 (16.3%) | 42 (22.8%) | 22 (22.0%) | 19 (17.8%) | 3 (12.0%) | |
| 3 | 81 (10.2%) | 35 (9.3%) | 21 (11.4%) | 12 (12.0%) | 10 (9.3%) | 3 (12.0%) | |
| Split-dose cisplatin | | | | | | | p=0.007 |
| 1 | 206 (26.0%) | 94 (25.1%) | 41 (22.3%) | 34 (34.0%) | 31 (29.0%) | 6 (24.0%) | |
| 2 | 288 (36.4%) | 135 (36.0%) | 64 (34.8%) | 34 (34.0%) | 46 (43.0%) | 9 (36.0%) | |
| 3 | 108 (13.7%) | 48 (12.8%) | 40 (21.7%) | 8 (8.0%) | 7 (6.5%) | 5 (20.0%) | |
| Carboplatin | | | | | | | p=0.108 |
| 1 | 98 (12.4%) | 53 (14.1%) | 22 (12.0%) | 10 (10.0%) | 11 (10.3%) | 2 (8.0%) | |
| 2 | 194 (24.5%) | 106 (28.3%) | 38 (20.7%) | 18 (18.0%) | 25 (23.4%) | 7 (28.0%) | |
| 3 | 261 (33.0%) | 118 (31.5%) | 52 (28.3%) | 44 (44.0%) | 38 (35.5%) | 9 (36.0%) | |
| Single-agent IO | | | | | | | p=0.027 |
| 1 | 28 (3.5%) | 12 (3.2%) | 11 (6.0%) | 3 (3.0%) | 2 (1.9%) | 0 (0%) | |
| 2 | 70 (8.8%) | 28 (7.5%) | 23 (12.5%) | 12 (12.0%) | 5 (4.7%) | 2 (8.0%) | |
| 3 | 111 (14.0%) | 64 (17.1%) | 22 (12.0%) | 8 (8.0%) | 13 (12.1%) | 4 (16.0%) | |
| EV+P | | | | | | | p=0.023 |
| 1 | 99 (12.5%) | 33 (8.8%) | 45 (24.5%) | 15 (15.0%) | 5 (4.7%) | 1 (4.0%) | |
| 2 | 37 (4.7%) | 15 (4.0%) | 8 (4.3%) | 9 (9.0%) | 2 (1.9%) | 3 (12.0%) | |
| 3 | 108 (13.7%) | 47 (12.5%) | 31 (16.8%) | 14 (14.0%) | 16 (15.0%) | 0 (0%) | |
| Other chemotherapy | | | | | | | p=0.471 |
| 1 | 12 (1.5%) | 8 (2.1%) | 2 (1.1%) | 0 (0%) | 2 (1.9%) | 0 (0%) | |
| 2 | 42 (5.3%) | 25 (6.7%) | 8 (4.3%) | 4 (4.0%) | 5 (4.7%) | 0 (0%) | |
| 3 | 88 (11.1%) | 50 (13.3%) | 11 (6.0%) | 10 (10.0%) | 14 (13.1%) | 3 (12.0%) | |
| **Consider a patient with mUC, a creatinine clearance of 50-55 ml/min, ECOG PS = 0-1, and no other comorbidities affecting treatment decision. Which regimen(s) would be your first, second, and third choice for mUC first-line treatment?** | | | | | | | |
| Standard-dose cisplatin | | | | | | | p=0.446 |
| 1 | 242 (30.6%) | 118 (31.5%) | 57 (31.0%) | 21 (21.0%) | 38 (35.5%) | 8 (32.0%) | |
| 2 | 153 (19.3%) | 66 (17.6%) | 35 (19.0%) | 20 (20.0%) | 23 (21.5%) | 9 (36.0%) | |
| 3 | 110 (13.9%) | 52 (13.9%) | 26 (14.1%) | 16 (16.0%) | 13 (12.1%) | 3 (12.0%) | |
| Split-dose cisplatin | | | | | | | p=0.157 |
| 1 | 273 (34.5%) | 140 (37.3%) | 48 (26.1%) | 36 (36.0%) | 39 (36.4%) | 10 (40.0%) | |
| 2 | 248 (31.4%) | 106 (28.3%) | 68 (37.0%) | 31 (31.0%) | 39 (36.4%) | 4 (16.0%) | |
| 3 | 112 (14.2%) | 56 (14.9%) | 29 (15.8%) | 9 (9.0%) | 12 (11.2%) | 6 (24.0%) | |
| Carboplatin | | | | | | | p=0.165 |
| 1 | 130 (16.4%) | 64 (17.1%) | 20 (10.9%) | 19 (19.0%) | 22 (20.6%) | 5 (20.0%) | |
| 2 | 217 (27.4%) | 125 (33.3%) | 41 (22.3%) | 19 (19.0%) | 26 (24.3%) | 6 (24.0%) | |
| 3 | 211 (26.7%) | 88 (23.5%) | 51 (27.7%) | 34 (34.0%) | 33 (30.8%) | 5 (20.0%) | |
| Single-agent IO | | | | | | | p=0.885 |
| 1 | 29 (3.7%) | 15 (4.0%) | 8 (4.3%) | 3 (3.0%) | 2 (1.9%) | 1 (4.0%) | |
| 2 | 75 (9.5%) | 42 (11.2%) | 13 (7.1%) | 13 (13.0%) | 6 (5.6%) | 1 (4.0%) | |
| 3 | 151 (19.1%) | 77 (20.5%) | 35 (19.0%) | 19 (19.0%) | 17 (15.9%) | 3 (12.0%) | |
| EV+P | | | | | | | p=0.001 |
| 1 | 96 (12.1%) | 27 (7.2%) | 45 (24.5%) | 20 (20.0%) | 4 (3.7%) | 0 (0%) | |
| 2 | 45 (5.7%) | 14 (3.7%) | 16 (8.7%) | 10 (10.0%) | 2 (1.9%) | 3 (12.0%) | |
| 3 | 94 (11.9%) | 43 (11.5%) | 26 (14.1%) | 10 (10.0%) | 12 (11.2%) | 3 (12.0%) | |
| Other chemotherapy | | | | | | | p=0.286 |
| 1 | 20 (2.5%) | 11 (2.9%) | 6 (3.3%) | 1 (1.0%) | 2 (1.9%) | 0 (0%) | |
| 2 | 43 (5.4%) | 19 (5.1%) | 11 (6.0%) | 5 (5.0%) | 8 (7.5%) | 0 (0%) | |
| 3 | 78 (9.9%) | 44 (11.7%) | 11 (6.0%) | 8 (8.0%) | 13 (12.1%) | 2 (8.0%) | |

| | Overall<br><br>N=791 | Europe[a]<br><br>N=375 | North America[b]<br>N=184 | Brazil<br><br>N=100 | India<br><br>N=107 | Australia<br><br>N=25 | *P value* |
|---|---|---|---|---|---|---|---|
| **Consider a patient with mUC, a creatinine clearance of 45-50 ml/min, ECOG PS = 0-1, and no other comorbidities affecting treatment decision. Which regimen(s) would be your first, second, and third choice for mUC first-line treatment?** | | | | | | | |
| Standard-dose cisplatin | | | | | | | p=0.105 |
| 1 | 251 (31.7%) | 128 (34.1%) | 61 (33.2%) | 20 (20.0%) | 33 (30.8%) | 9 (36.0%) | |
| 2 | 101 (12.8%) | 39 (10.4%) | 27 (14.7%) | 12 (12.0%) | 19 (17.8%) | 4 (16.0%) | |
| 3 | 109 (13.8%) | 48 (12.8%) | 23 (12.5%) | 18 (18.0%) | 17 (15.9%) | 3 (12.0%) | |
| Split-dose cisplatin | | | | | | | p=0.214 |
| 1 | 201 (25.4%) | 93 (24.8%) | 41 (22.3%) | 23 (23.0%) | 36 (33.6%) | 8 (32.0%) | |
| 2 | 287 (36.3%) | 138 (36.8%) | 70 (38.0%) | 32 (32.0%) | 38 (35.5%) | 9 (36.0%) | |
| 3 | 109 (13.8%) | 51 (13.6%) | 31 (16.8%) | 13 (13.0%) | 11 (10.3%) | 3 (12.0%) | |
| Carboplatin | | | | | | | p=0.008 |
| 1 | 196 (24.8%) | 107 (28.5%) | 24 (13.0%) | 34 (34.0%) | 24 (22.4%) | 7 (28.0%) | |
| 2 | 202 (25.5%) | 97 (25.9%) | 45 (24.5%) | 24 (24.0%) | 29 (27.1%) | 7 (28.0%) | |
| 3 | 203 (25.7%) | 93 (24.8%) | 53 (28.8%) | 25 (25.0%) | 28 (26.2%) | 4 (16.0%) | |
| Single-agent IO | | | | | | | p=0.630 |
| 1 | 29 (3.7%) | 14 (3.7%) | 6 (3.3%) | 4 (4.0%) | 5 (4.7%) | 0 (0%) | |
| 2 | 100 (12.6%) | 57 (15.2%) | 20 (10.9%) | 14 (14.0%) | 7 (6.5%) | 2 (8.0%) | |
| 3 | 144 (18.2%) | 71 (18.9%) | 37 (20.1%) | 17 (17.0%) | 14 (13.1%) | 5 (20.0%) | |
| EV+P | | | | | | | p<0.001 |
| 1 | 95 (12.0%) | 23 (6.1%) | 47 (25.5%) | 19 (19.0%) | 6 (5.6%) | 0 (0%) | |
| 2 | 52 (6.6%) | 20 (5.3%) | 14 (7.6%) | 14 (14.0%) | 3 (2.8%) | 1 (4.0%) | |
| 3 | 92 (11.6%) | 50 (13.3%) | 22 (12.0%) | 7 (7.0%) | 10 (9.3%) | 3 (12.0%) | |
| Other chemotherapy | | | | | | | p=0.575 |
| 1 | 17 (2.1%) | 10 (2.7%) | 5 (2.7%) | 0 (0%) | 2 (1.9%) | 0 (0%) | |
| 2 | 36 (4.6%) | 22 (5.9%) | 6 (3.3%) | 3 (3.0%) | 5 (4.7%) | 0 (0%) | |
| 3 | 96 (12.1%) | 49 (13.1%) | 11 (6.0%) | 16 (16.0%) | 16 (15.0%) | 4 (16.0%) | |

EV+P, enfortumab vedotin + pembrolizumab; IO, immunotherapy; la/mUC, locally advanced or metastatic urothelial cancer.

[a] Europe includes respondents from France, Germany, Italy, Spain, and the UK. [b] North America includes respondents from USA and Canada.

**Supplemental Table S10. Split-dose cisplatin as a treatment option for otherwise fit patients with la/mUC and borderline kidney function.**

| | **N=791** |
|---|---|
| **How strongly do you agree with the following statement: "Split-dose cisplatin regimens are a reasonable treatment option for patients with la/mUC and a creatinine clearance of 45-55 mL/min, ECOG PS 0-1, and no other comorbidities affecting treatment decision."** | |
| Strongly agree | 167 (21.1) |
| Agree | 449 (56.8) |
| Neither agree nor disagree | 122 (15.4) |
| Disagree | 29 (3.7) |
| Strongly disagree | 6 (0.8) |
| Unsure | 18 (2.3) |

ECOG PS, Easter Cooperative Oncology Group performance status; la/mUC, locally advanced or metastatic urothelial cancer.

**Supplemental Figure S1. Comparison of split-dose cisplatin to other regimens in otherwise fit patients with locally advanced or metastatic urothelial cancer and a creatinine clearance of 45 to 55 mL/min.**

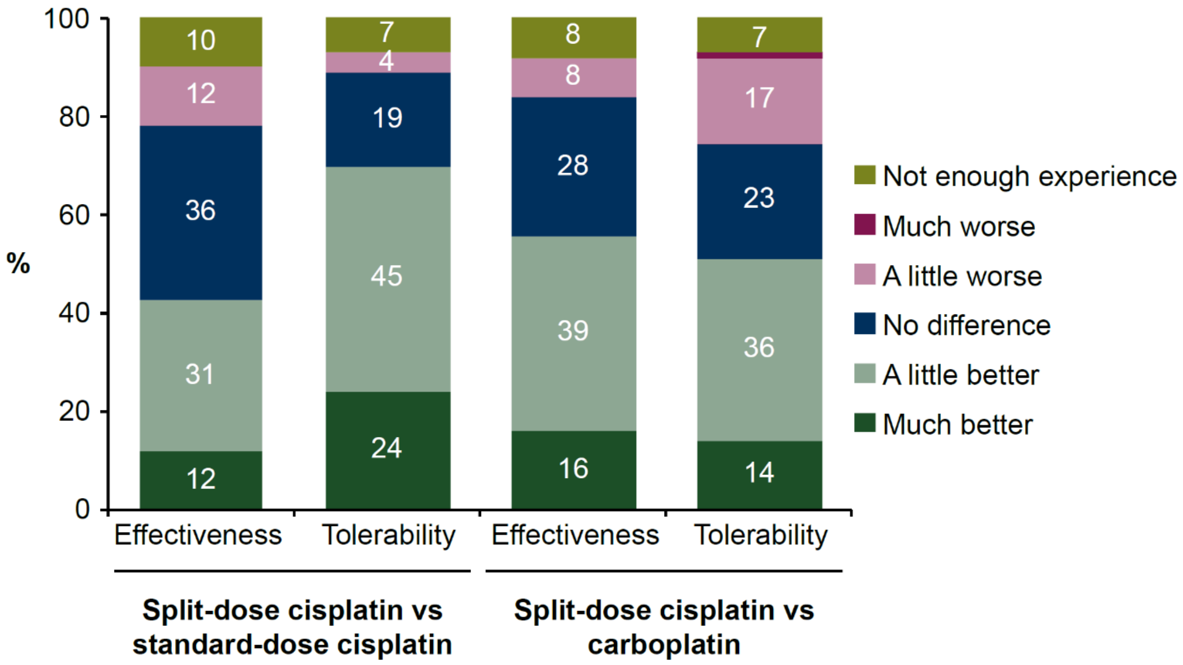

Supplement: Supplementary file 1 [file cancers-17-00509-s001.zip › cancers-3352714-supplementary.pdf]
